# Digital Microneedles for Multiplexed Transdermal Sensing via Fluorescent QR Codes

**DOI:** 10.1002/adma.202518935

**Published:** 2026-03-10

**Authors:** Farbod Abazar, Shahrokh Vahabi, Elena Bellotti, Martina Corsi, Irene Nocera, Paola Orlandi, Guido Bocci, Giuseppe Barillaro

**Affiliations:** ^1^ Information Engineering Department University of Pisa Pisa Italy; ^2^ Department of Veterinary Sciences University of Pisa Pisa Italy; ^3^ Department of Clinical and Experimental Medicine School of Medicine University of Pisa Pisa Italy; ^4^ Department of Translational Research and New Technologies In Medicine and Surgery School of Medicine University of Pisa Pisa Italy

**Keywords:** digital biosensing, fluorescent microneedles, QR code, transdermal health monitoring

## Abstract

Real‐time biochemical sensing is essential for precision medicine, yet current wearable and transdermal biosensors suffer from signal drift, calibration demands, and limited multiplexing capabilities in vivo. Here, we introduce digital fluorescent microneedles that translate analyte concentrations into scannable QR codes via threshold‐activated probes. Each microneedle functions as a fluorescent binary switch, turning “on” only above a defined analyte threshold, thereby eliminating the need for calibration and enhancing robustness against tissue heterogeneity and environmental noise. The microneedles feature a biodegradable, mechanically optimized “baby‐bottle” design that enables reliable skin penetration and controlled tip detachment. Central to this concept is the rational engineering of fluorescent probes with discrete activation thresholds, which—when embedded in microneedles—produce stepwise readouts spanning physiopathological ranges of pH (4.5–8.5) and glucose (1–10 mM). By tuning probe loading, we achieve reproducible threshold activation, enabling fully digital, multiplexed detection of pH and glucose in skin with classification accuracies of 93% and 85%. This digital encoding concept is broadly extensible to diverse probes and biomarkers, providing a scalable route to calibration‐free, multiplexed biosensing in vivo. The QR‐based output delivers a direct, quantitative representation of biochemical information, facilitating decentralized diagnostics and integration into digital health workflows. Together, these advances establish digital microneedles as a versatile and clinically relevant platform for transdermal biosensing.

## Graphical abstract

Digital fluorescent microneedles encode biochemical information in skin into scannable QR codes through threshold‐activated probes that generate binary on/off signals. By transforming analog fluorescence into discrete digital patterns, this platform enables calibration‐free, multiplexed transdermal sensing of physiologically relevant biomarkers.

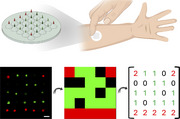

## 1 Introduction

The advent of wearable biosensors has transformed health monitoring by enabling real‐time tracking of physiological parameters through the body's natural biochemical signals. Among these, sweat‐based sensors have garnered significant attention due to their non‐invasiveness and ease of integration into daily life [1, 2]. However, despite their promise, sweat biosensors face fundamental limitations that restrict their accuracy and clinical utility. The low concentration of biomarkers in sweat, coupled with environmental variability (e.g., temperature, humidity, and secretion rate fluctuations), introduces substantial signal noise and inconsistencies, reducing both sensitivity and specificity. Moreover, the episodic nature of sweat secretion leads to discontinuous data collection, making it unsuitable for continuous, high‐fidelity health monitoring.

As precision healthcare advances, there is a growing demand for minimally invasive, multiplexed, and scalable biosensing technologies capable of monitoring diverse biomarkers with high accuracy and reproducibility in real time. Dermal sensing of extracellular fluid (ECF) with microneedles has emerged as a minimally invasive, promising alternative, offering direct access to a stable and information‐rich biofluid [3, 4]. Unlike sweat, ECF maintains a dynamic yet consistent biomarker profile, allowing for high‐fidelity, real‐time sensing with improved specificity. Furthermore, as ECF resides within the protective barrier of the skin, it is largely insulated from external contaminants, ensuring a robust and reliable data stream unaffected by environmental fluctuations.

Microneedles, originally developed for transdermal drug delivery [5, 6], have been adapted for biosensing, leveraging their ability to penetrate the skin painlessly and access interstitial biofluids with minimal discomfort [7]. Current microneedle biosensors primarily rely on electrochemical detection methods [8] and, more recently, optical approaches, such as colorimetric [9] and fluorescence‐based readouts [10]. While these technologies have improved biosensing capabilities, existing microneedle‐based platforms remain constrained by several key limitations. Most designs rely on a supporting substrate that remains attached to the skin after penetration, restricting their pairing with wearable readout electronics for prolonged monitoring within the skin [11]. Additionally, most existing microneedle‐based biosensors are designed for the detection of a single analyte—typically glucose or proteins—while their potential for multiplexed sensing remains underexplored.

Efforts to develop multi‐analyte microneedle biosensors have led to the introduction of colorimetric microneedle sensors capable of detecting pH, glucose, uric acid, and temperature at the dermal level [12]. However, colorimetric sensing suffers from poor sensitivity due to low contrast and difficulty in resolving small color changes through the skin. The reliance on a precise and often nonlinear in vitro calibration further complicates translation to in vivo applications. Fluorescence‐based microneedles leveraging analog intensity measurements have been introduced as an alternative, offering higher sensitivity and wireless readout potential [13, 14]. These analog fluorescent microneedles detect analyte concentrations through graded changes in fluorescence intensity, ideally calibrated to yield a linear or quasi‐linear response curve. This curve allows users to infer concentration levels from intermediate fluorescence values. However, in practical scenarios, this intensity‐based readout is inherently vulnerable to numerous sources of variability—including differences in microneedle insertion depth, tissue scattering, fluorophore diffusion, and environmental lighting—leading to poor reproducibility, calibration drift, and limited reliability in vivo. Moreover, analog readouts require finely tuned calibration curves that must be adjusted for each application and often fail to generalize across users or conditions, especially in continuous or long‐term monitoring scenarios where signal drift and degradation can further obscure interpretation.

Here, we introduce digital fluorescent microneedles that encode biochemical information into discrete, binary fluorescence states (“on”/“off”), rather than inferring analyte levels from analog intensity values. Each microneedle functions as a thresholding element with a sharp, nonlinear switching response, converting local chemistry into a binary decision that eliminates the need for in vivo calibration and mitigates variability from tissue optics and insertion depth, key limitations of conventional analog fluorescent microneedles. By arranging the microneedles into 2D arrays, analyte levels are digitized into spatial codes, producing scannable QR patterns with built‐in fault tolerance and straightforward scalability to multiplexed sensing. We validate this concept through simultaneous detection of pH and glucose in skin under physiopathological conditions, achieving real‐time, robust QR‐code readout with classification accuracies of 93% and 85%, respectively. Just as digital electronics revolutionized analog computation, digital microneedles establish a new paradigm for calibration‐free, multiplexed, and clinically actionable transdermal biosensing, with broad implications for wearable diagnostics, decentralized monitoring, and personalized medicine.

## 2 Results and Discussion

**FIGURE 1:**
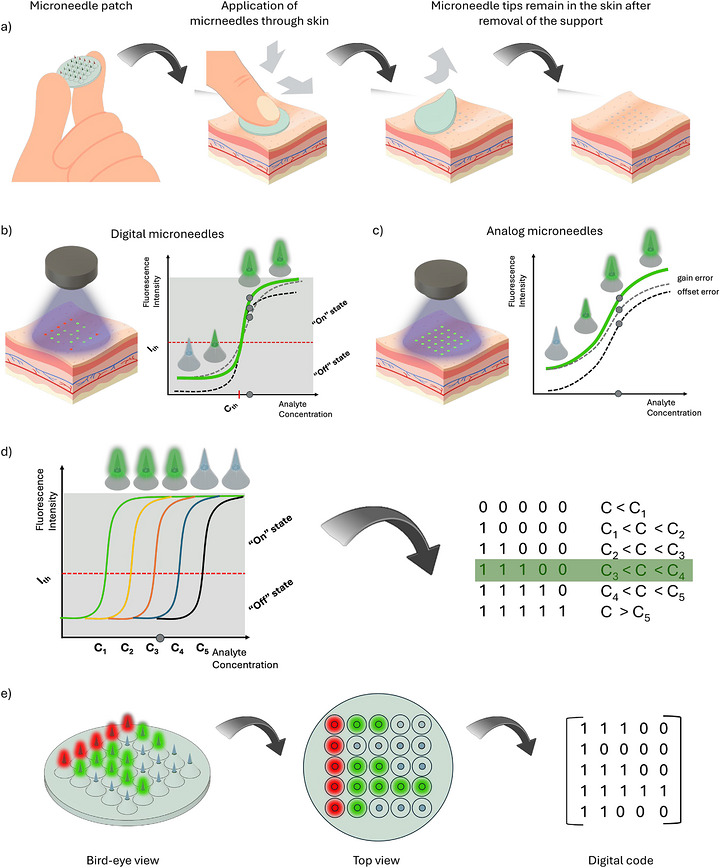
Conceptual framework contrasting analog vs. digital fluorescent microneedle sensing. (a,b) Schematic workflow: patch application and tip detachment after skin insertion (a), followed by optical readout by blue‐light illumination producing a scannable QR code that dynamically reflects analyte concentrations (b). Digital microneedles in (b) exhibit sharp, nonlinear activation at defined thresholds, functioning as binary switches that eliminate calibration and reduce ambiguity. (c) Analog microneedles, in contrast to digital ones, generate graded fluorescence intensity as a function of analyte concentration, requiring calibration and remaining prone to variability from tissue and environmental conditions, leading to gain and offset errors. (d) Staggered thresholds across multiple microneedles generate binary fluorescent codes corresponding to analyte concentration ranges. (e) Arrangement of digital microneedles in 2D arrays produces fluorescent QR codes whose patterns dynamically change with analyte levels, enabling direct optical decoding of biochemical information. Green fluorescence indicates sensing microneedles, while red fluorescence marks reference microneedles for orientation and insertion confirmation.

Figure 1 illustrates the workflow and conceptual framework of multianalyte sensing with digital fluorescent microneedles. After application, the patch penetrates the skin, and the microneedle tips remain embedded following patch removal (Figure 1a). Each microneedle incorporates a fluorescent probe responsive to a specific analyte, producing green fluorescence above a defined concentration threshold (“on” state) under blue‐light excitation (Figure 1b). Figure 1b,c highlight the fundamental distinction between analog and digital microneedles: whereas analog devices generate graded fluorescence signals that are susceptible to variability and calibration drift (Figure 1c), digital microneedles exhibit sharp, nonlinear activation at preset thresholds, enabling unambiguous binary outputs.

This threshold‐based behavior can be tuned by adjusting probe concentrations across microneedles within the array. As shown in Figure 1d, staggered response curves allow distinct analyte ranges to be resolved, each encoded as an “on” or “off” state. By incorporating probes for different biomarkers within the same array, simultaneous multiplexed detection becomes possible. Figure 1e illustrates how these binary states are spatially organized into two‐dimensional scannable QR‐code patterns that dynamically adapt to changes in biochemical conditions. These digital outputs provide an interpretable, fault‐tolerant, and calibration‐free readout suitable for real‐time sensing, diagnostics, and programmable feedback. Reference red dyes with concentration‐independent emission confirm microneedle insertion and provide orientation markers for accurate optical decoding.

Compared to conventional staining dyes, fluorescent probes offer key advantages for microneedle‐based biosensing [15]. Fluorescent materials emit bright, well‐defined signals upon specific excitation, ensuring visibility and reliable scanning under low‐light conditions. Distinct emission ranges (e.g., red, green, blue) enable multichannel encoding, while high signal‐to‐noise ratios improve readability on heterogeneous substrates such as skin. Fluorescent probes are also more durable and stable than dyes, and many are inherently, or can be engineered to be, responsive to environmental stimuli such as pH, temperature, or biomolecule concentration, ensuring robust and tamper‐proof information encoding [16, 17].

**FIGURE 2:**
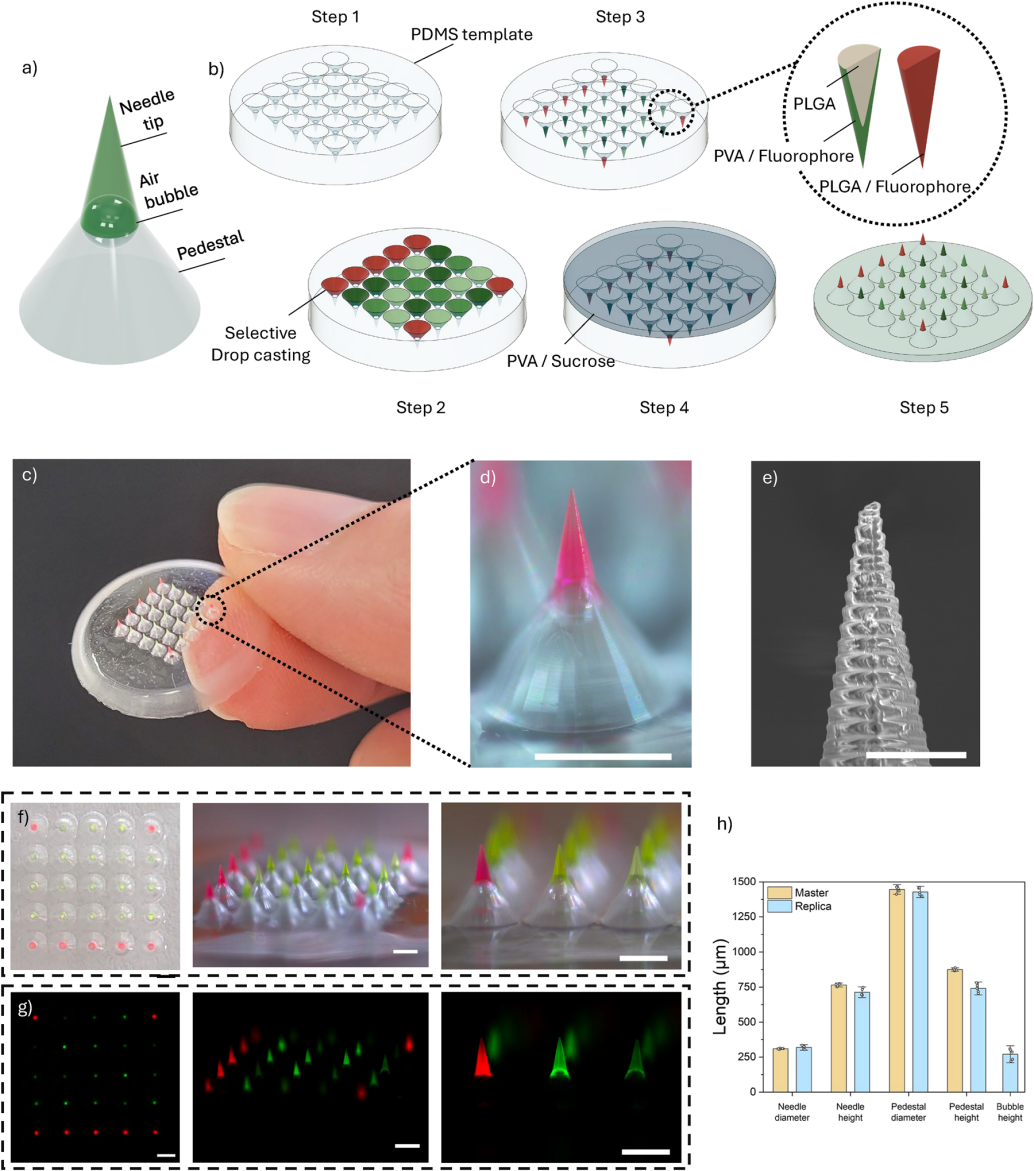
Fabrication and morphological characterization of digital QR code microneedles. (a) Schematic of the needle architecture. (b) Key fabrication steps: Step 1, production of a negative PDMS mold. Step 2, selective drop‐casting of PVA/green‐fluorescent‐probe solution for fabrication of core–shell sensing needles; Step 3, centrifugation to form a thin hydrophilic PVA shell, subsequently backfilled with a PLGA core (inset). Reference needles are fabricated by drop‐casting PLGA solution containing red dye in designed cavities, with centrifugation ensuring fully filled tips (inset). Step 4, casting of a PVA–sucrose pedestal and backing layer. Step 5, demolding of the completed patch. (c,d) Photograph of a QR code microneedle patch featuring polymer tips loaded with red (reference) and green (sensitive) fluorophores (c), and close‐up view of one of the microneedles in the array (d) (scale bar, 1 mm). e) SEM close‐up of an individual microneedle tip showing precise geometry (scale bar, 20 µm). (f,g) Top and bird's‐eye views of the patch in (c) under bright‐field (f) and fluorescence (g) modes; (scale bars, 1 mm). (h) Quantitative morphological analysis confirming the reliability and consistency of the fabrication process (*n* = 5 patches). Data in h) are reported as mean ± standard deviation (SD) from independent experiments.

The fabrication process of microneedle patches is shown in Figure 2b,c. The patch consists of a 5 × 5 array of microneedles with a spacing of 1.5 mm and a distinctive “baby‐bottle” shape, designed to enable effective skin penetration and controlled post‐application separation (Figure 2b). Each microneedle features a narrow conical tip to facilitate insertion and a wider pedestal‐like base that provides structural support and enhances insertion depth. The tip adopts a core–shell design that decouples analyte access and probe interaction in the hydrated shell from the mechanical robustness of the core, enabling independent optimization of digital sensing performance and insertion/detachment reliability. The inclusion of an air bubble between the tip and pedestal preserves mechanical integrity under compression while weakening the structure under shear stress, enabling smooth tip detachment after skin insertion. The patch is designed for single use, as the detached microneedle tips are made of biodegradable materials that subsequently resorb.

Figure 2a,b outlines the architecture and main fabrication steps of the digital microneedles, respectively. First, a negative PDMS mold is produced by replica molding a high‐resolution 3D‐printed master of the baby‐bottle‐shaped microneedles (Step 1, with the master shown in Figure S1a–c). Core–shell microneedles for digital sensing are prepared by selectively drop‐casting a PVA solution doped with green fluorescent probes into the mold cavities (Step 2). After centrifugation (Step 3), a thin hydrophilic PVA shell forms along the tip walls and is subsequently backfilled with a PLGA core (inset, Step 3). Reference needles are fabricated in parallel by loading a PLGA solution containing a red fluorescent dye into designated cavities, which, after centrifugation, fully fills the conical tips, minimizing porosity and enhancing mechanical strength. Next, an aqueous PVA–sucrose solution is cast to form the pedestal and the patch backing (Step 4). The surface tension of the PVA traps an air bubble between the dried PLGA tip and the pedestal [18]. After drying, the patch is peeled from the mold (Step 5). The final device is a 1.5‐cm‐diameter circular patch, user‐friendly and mechanically robust, which can be applied by simply pressing it onto the skin. All materials used are biodegradable and biocompatible [19], supporting safe in vivo use and environmental sustainability.

A prototype QR code microneedle patch is shown in Figure 2c, where the needle tips encode a digital pattern using green fluorescein‐based probes for sensing needles and Nile Red dyes for reference needles. The inset of Figure 2d highlights the microneedle architecture, showing the confined fluorescent tip, separated from the water‐soluble pedestal by an air bubble. No cross‐contamination is observed between adjacent needles or between the tips and the pedestal. A close‐up of tip morphology is provided in Figure 2e. The patch is further visualized in Figure 2f,g, which presents bright‐field and fluorescence images from top and angled perspectives. Upon blue‐light excitation, the fluorophores embedded in the needle tips emit light, producing a scannable QR code. This fabrication method is highly versatile, enabling encoding of arbitrary single‐ or multi‐fluorophore patterns within the microneedles, as demonstrated in Figure S2.

We systematically evaluated the reliability of the microneedle fabrication process across multiple patches, with results summarized in Figure 2h. The microneedles exhibited excellent shape fidelity and dimensional consistency when compared to the original master mold (Figure S1d–f). Importantly, this consistency was maintained even in the presence of the embedded air bubble between the pedestal and needle tip. The fabrication process demonstrated a maximum coefficient of variation (CV%) of 4.3%, highlighting the high reproducibility and robustness of the microneedle patch production method.

**FIGURE 3:**
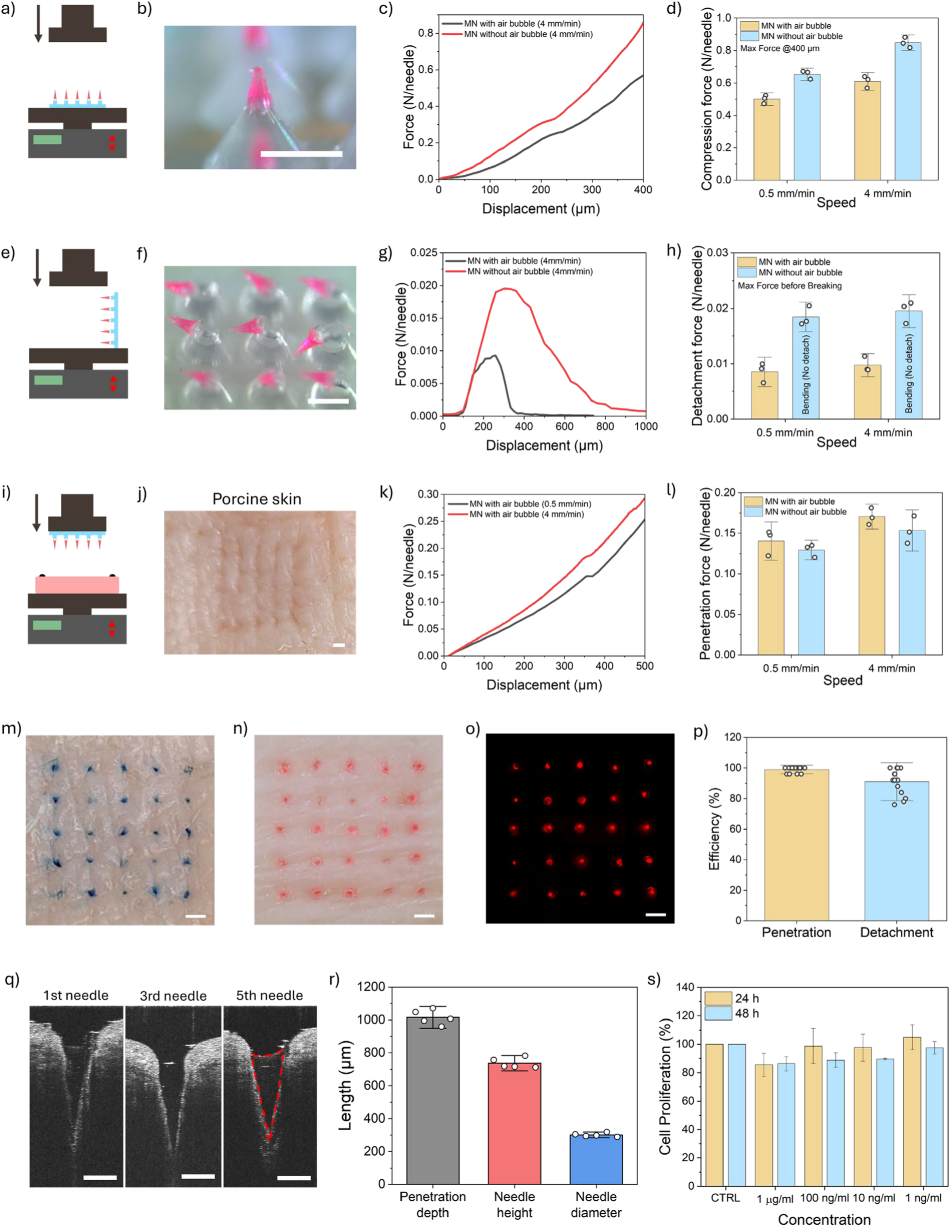
Mechanical performance and skin penetration of QR code microneedle patches. (a–d) Compression testing: schematic setup (a), optical image of a loaded microneedle (b, scale bar 1 mm), force–displacement curves comparing microneedles with (red trace) and without (black trace) embedded air bubble (c), and quantitative analysis of the compression force after a travel of 400 µm (*n* = 3 patches) (d). Bubble‐containing microneedles showed slightly reduced rigidity, consistent with bubble deformation. (e–h) Shear testing: schematic setup (e), optical image of microneedle detachment under shear (f, scale bar 1 mm), representative force–displacement curves (g), and quantitative analysis of the detachment force (*n* = 3 patches) (h). Detachment occurred at ∼0.01 N per needle for bubble‐containing microneedles, within the range of manual thumb force, while controls bent without detachment. (i–l) Penetration testing: schematic setup (i), porcine skin after microneedle insertion (j, scale bar 1 mm), force–displacement curve showing penetration at ∼0.15 N per needle (k), and statistical analysis of the penetration force at different insertion speeds (*n* = 3 patches) (l). (m–p) Insertion and detachment efficiency in porcine skin: dye staining of penetration sites (m), bright‐field (n) and fluorescence (o) images of embedded tips after detachment of the patch support, and quantitative penetration and detachment efficiencies (p). Results show penetration and detachment efficiencies of 98% and 91%, respectively (*n* = 12 patches); (m‐o, scale bar is 1 mm). (q) OCT cross‐sectional images of three microneedle tips (of a single row) in porcine skin after shear‐assisted deployment. The red dashed line highlights the needle tips within the skin (*n* = 3 needles). Scale bar, 300 µm. (r) Quantification of microneedle tip geometry and insertion depth in porcine skin obtained from OCT images (*n* = 5 needles). (s) Proliferation of HDNF cells treated with microneedle extracts at different concentrations for 24 and 48 h. Cell proliferation is expressed as a percentage relative to the control. Data in (d), (h), (l), (p), (r) are reported as mean ± standard deviation (SD) from independent experiments. Data in (s) are reported as mean ± standard deviation (SD) from three independent wells per each concentration. Statistical analysis was performed using one‐way ANOVA followed by post hoc multiple‐comparison tests; significance (*p* < 0.05) was observed only at 1 µg mL^−^^1^ after 48 h.

We next evaluated the mechanical performance of the microneedle patch under compression and shear force tests to assess its ability to penetrate the skin and enable tip detachment post‐application (Figure 3). Microneedle patches composed of solid PLGA without an embedded air bubble were used as controls to isolate the effect of the air bubble on mechanical behavior. Under uniaxial compression against a stainless‐steel plate, both control and air bubble–containing microneedles exhibited a steady increase in force with displacement (Figure 3a–d). Microneedles with air bubbles showed slightly reduced rigidity, consistent with bubble deformation, which lowered the effective force transmitted to the tip by ∼0.2 N per needle. At a compression force of ∼0.25 N per needle, plastic deformation of the tip was observed (Figure 3b–d; Figure S3e). Shear force testing revealed that microneedles with embedded air bubbles detached readily from the pedestal, as indicated by a sudden drop in the force–displacement curve (Figure 3e–h). Detachment occurred at ∼0.01 N per needle, well within the range of manual thumb application (Figure 3f–h). In contrast, control microneedles without an air bubble did not detach under shear stress, instead bending or deforming even at higher applied forces (Figure S3f).

We further evaluated patch functionality through penetration tests in porcine skin (Figure 3i–l). Ethical compliance: porcine skin samples were obtained from animals euthanized for independent experimental purposes at the University of Pisa. As no animals were sacrificed specifically for this study and tissues were reused in accordance with the 3Rs principles (Replacement, Reduction, and Refinement), ethical approval was not required under applicable institutional and national regulations. The force–displacement curve exhibited a clear inflection after ∼400 µm of travel, corresponding to successful skin entry at a force of ∼0.15 N per needle—lower than the force required to cause tip deformation (Figure 3j–l). Penetration was validated by tissue staining, which confirmed that the needle tips crossed the skin surface. OCT cross‐sectional imaging further confirmed that, following shear‐assisted deployment, only the conical microneedle tips remained implanted in porcine skin, with implantation depth and tip geometry consistent with the design values (tip height ≈800 µm and base radius ≈160 µm; Figure 3q,r). Biosafety of the microneedle system was assessed by in vitro cytotoxicity using normal human dermal fibroblasts (NHDFs) exposed to microneedle‐conditioned (extract) media. As shown in Figure 3s, cell proliferation after 24 and 48 h remained comparable to the control across all tested extract concentrations. Representative optical micrographs (Figure S4) further confirmed preserved cell morphology and confluency, indicating no detectable cytotoxic effects attributable to the microneedles or the loaded probes.

To evaluate microneedle patch performance under operational conditions, we conducted insertion experiments using manual thumb‐applied force to mimic practical deployment (Figure 3m–p). Patches with needle tips loaded with Nile Red were applied to porcine skin to enable post‐insertion fluorescence visualization. To decouple penetration and detachment, we performed independent assessments of each parameter. Penetration efficiency was quantified by applying and removing the microneedle patch without shear, followed by staining the skin to visualize insertion sites (Figure 3m). Detachment efficiency was assessed by applying shear force with the thumb after penetration. Fluorescence imaging of the skin post‐removal revealed embedded needle tips, confirming detachment (Figure 3n,o). A summary of results is shown in Figure 3p, demonstrating penetration and detachment efficiencies of 98% and 91%, respectively. These findings highlight the reliability and user‐friendliness of the patch for transdermal application under realistic conditions.

We next developed a dynamic, digital fluorescent QR code microneedle platform for real‐time transdermal biosensing. As a proof‐of‐concept, we targeted the simultaneous detection of glucose and pH, a clinically relevant combination since fluctuations in these parameters provide critical insight into metabolic health [20], wound healing [21], and diabetes monitoring [22]. Specifically, pH changes can indicate inflammation or infection, while glucose levels are closely linked to energy metabolism and glycemic control.

For this application, we designed a QR code microneedle array using fluorescein (FL) as a pH‐sensitive probe and fluorescein boronic acid (FLB) as a glucose‐sensitive probe, with Nile Red as a reference fluorophore to define QR code orientation. Upon insertion, the PVA shell rapidly hydrates, forming a thin, water‐swollen polymer network that allows diffusion of small analytes such as H^+^ ions and glucose into the shell, where they interact with fluorescent probes. The fluorescence intensity of FL increases with rising pH, whereas that of FLB decreases with increasing glucose concentration (Figure S5). The fluorescence signal equilibrates ∼30 min after microneedle insertion, regardless of analyte concentration and primarily governed by diffusion kinetics of the targets through the PVA shell encapsulating the probes (Figure S5).

**FIGURE 4:**
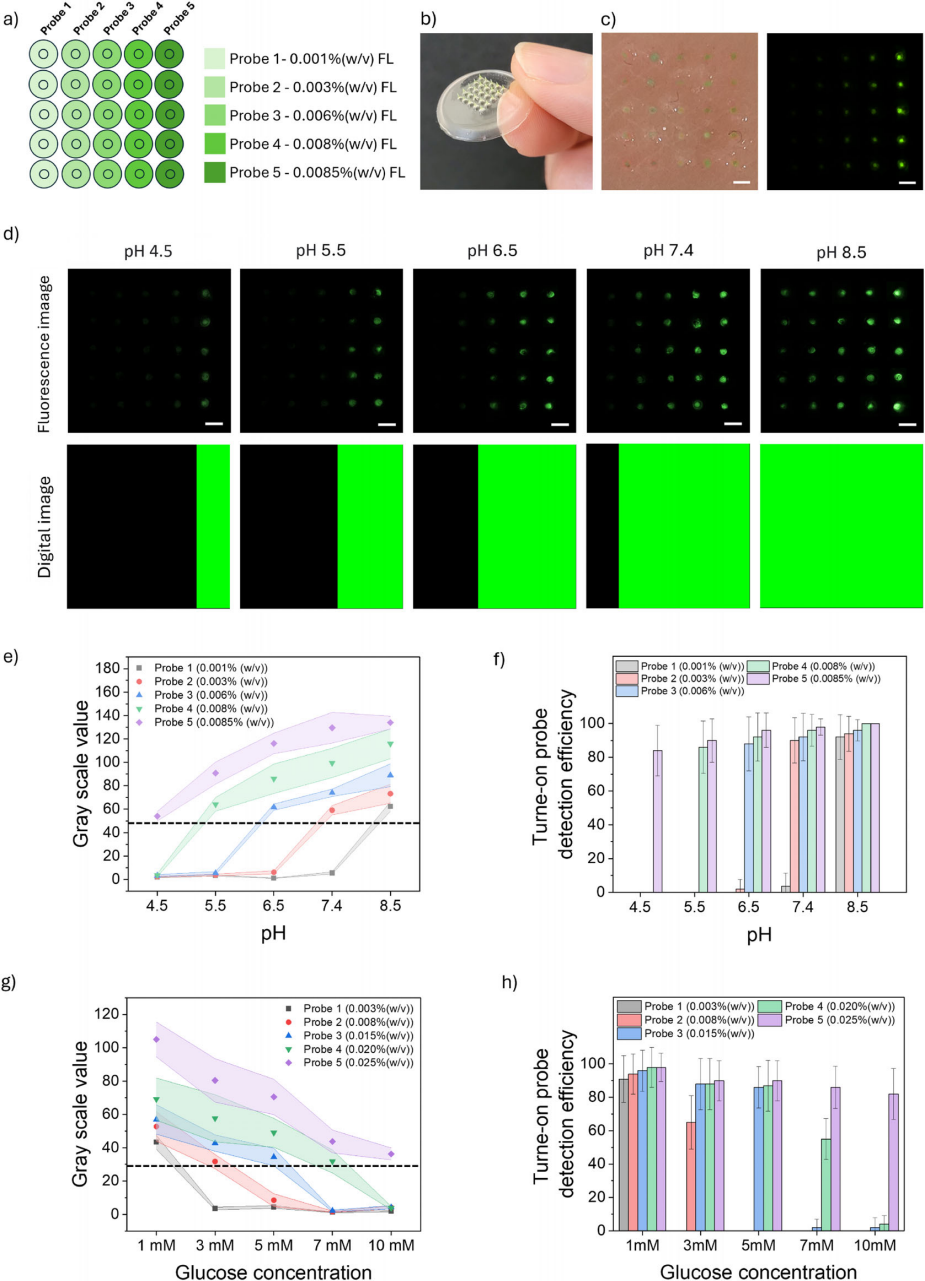
Optimization of threshold‐based fluorescent probes for digital sensing with microneedles. (a) Schematic top view of a 5 × 5 microneedle array containing fluorescein (FL) probes at different concentrations, each tuned to activate above specific pH thresholds (pH 4–8, in 1‐unit increments). Each column (five microneedles) was loaded with the same probe concentration to evaluate reproducibility. (b) Optical image of a fabricated microneedle patch. (c) Bright‐field and fluorescence images of the patch following insertion into synthetic skin (scale bar is 1 mm). (d) Fluorescence images of microneedle patches inserted into synthetic skin at different pH levels (pH 4.5–8.5), with corresponding binarized QR code outputs (scale bar is 1 mm). (e) Quantification of green‐channel fluorescence (Value, V) across columns. FL concentration defines the pH activation threshold, with “on” states consistently above V = 50 and “off” states below V = 10 (*n* = 5 patches). (f) Detection efficiency for each FL concentration, defined as the percentage of microneedles exceeding the threshold at each pH, confirming reliable and concentration‐specific activation (*n *= 5 patches). (g) Fluorescence response of microneedles loaded with fluorescein boronic acid (FLB) for glucose sensing, showing decreased fluorescence above probe‐specific glucose thresholds (2–8 mM, in 2 mM steps) in the range 1–10 mM (*n* = 5 patches). (h) Detection efficiency for FLB‐based microneedles confirming high reproducibility and effective threshold tuning (*n *= 5 patches). Data in (f) and (h) are reported as mean ± standard deviation (SD) from independent experiments.

To implement threshold‐based sensing, we first optimized the concentrations of FL and FLB within the PVA shell across the sensing needles. By adjusting probe concentration, activation thresholds were precisely aligned with specific analyte levels, detectable by a given optical system. This tuning strategy enabled a binary fluorescence response, where the probes remained “off” below a defined analyte concentration and turned “on” once the threshold was exceeded—for pH in the case of FL, and the reverse for glucose with FLB (Figure 4). Figure 4a shows a schematic top view of a microneedle patch in which each column was loaded with a different concentration of FL, tailored to activate above different pH thresholds ranging from pH 4 to 8 in 1‐unit increments. In wound care, clinically relevant pH shifts are often on the order of units, with open wounds typically reported in a neutral‐to‐alkaline range (∼pH 6.5–8.5) and chronic wounds frequently more alkaline (∼pH 7.2–8.9), making unit‐scale pH thresholding useful for physiopathological stratification [23]. Figure 4b shows the fabricated patch, and Figure 4c displays bright‐field and fluorescence images after insertion into the skin. Each column (five needles) contained identical probe concentration to evaluate turn‐on efficiency and reproducibility. To evaluate performance, patches were inserted into skin preconditioned overnight in PBS at pH 4.5 to 8.5 (1‐unit steps), and fluorescence images were captured 30 min post‐insertion, corresponding to probe equilibration time. A custom image‐processing script was developed to analyze fluorescence images by segmenting them into a 5 × 5 matrix, with each pixel corresponding to a microneedle. Images were processed in HSV (Hue, Saturation, Value) space, which decouples brightness from color. The algorithm evaluated the V value in the green channel and classified each needle as “on” or “off” according to a predefined threshold. These binary decisions were then mapped back into a matrix representation, forming a digitally encoded image (Figure S6). Figure 4d shows raw fluorescence images at different pH values, alongside corresponding digital outputs. The data reveal clear, threshold‐dependent activation of FL probes, with thresholds shifting as FL concentration in the PVA shell decreases. Although fluorescence intensity continues to rise beyond the threshold, this does not affect the digital “on/off” classification. Figure 4e quantifies the average green‐channel V values across columns vs. pH levels, showing distinct activation thresholds and low intra‐column variability. Activated needles consistently exceeded V = 50 (0–255 scale), while non‐activated needles remained below 10. This high contrast enables digital pH mapping with a resolution of 1 pH unit, corresponding to an intrinsic quantization uncertainty of ±0.5 pH, consistent with the defined pH thresholds. In this digital framework, resolution is indeed determined by the spacing of the encoded thresholds.

To assess sensing reliability, we calculated the detection efficiency for each probe, defined as the percentage of microneedles within a column exhibiting fluorescence intensities above the threshold at a given pH, averaged across multiple patches (Figure 4f). Detection efficiencies of 80%–100% confirmed high reproducibility with minimal false negatives.

To evaluate specificity and orthogonality, we conducted a parallel experiment using microneedles loaded with FLB probes for glucose detection (Figure S7). Each column was tuned to a distinct glucose threshold (2–8 mM, in 2 mM increments), allowing independent classification of activation. For glucose, our 2 mM thresholding corresponds to an intrinsic uncertainty of ±1 mM (≈±18 mg/dL), which is comparable in magnitude to the ±15 mg/dL criterion specified in ISO 15197:2013 for self‐monitoring blood glucose systems in the low‐glucose regime (<100 mg/dL) [24]; importantly, our objective here is calibration‐free, transdermal digital classification rather than ISO‐standard quantitative equivalence.

As shown in Figure 4g,h, FLB microneedles displayed a clear threshold‐dependent behavior: fluorescence remained high (>30) below the designated glucose level and dropped sharply (<10) once the threshold was exceeded. The discrete responses of each column enabled reliable differentiation of glucose concentrations with a resolution of 2 mM (uncertainty ±1 mM). Detection efficiency remained high across all thresholds and replicate patches (Figure 4h), confirming the robustness, reproducibility, and orthogonal multiplexing capability of the system.

**FIGURE 5:**
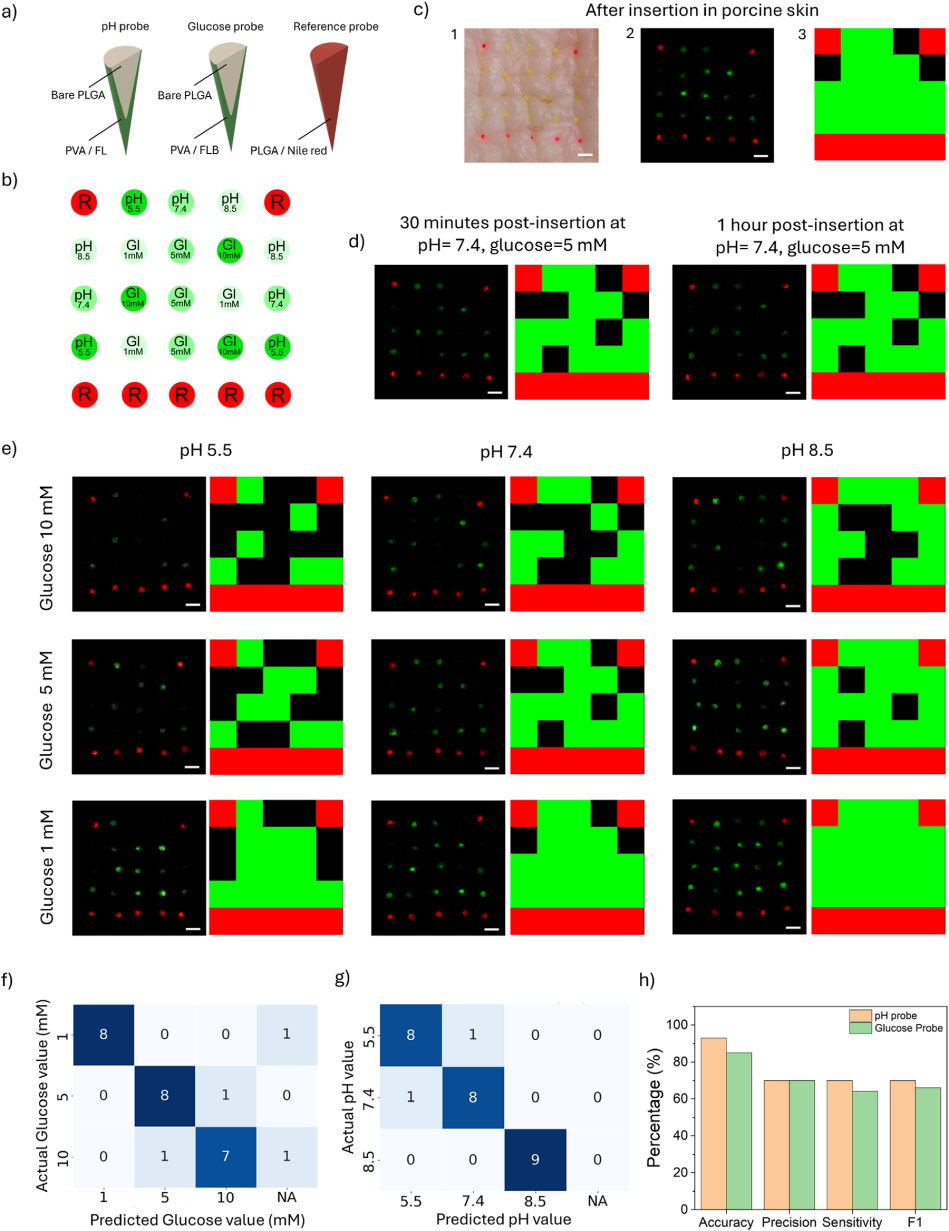
Characterization of dual‐analyte pH/glucose QR code microneedle patches in ISF incubated porcine skin. (a) Schematic of the microneedle core‐shell structure for pH/glucose detection and solid PLGA reference needles. (b) Patch layout with different fluorophore concentrations enabling dynamic threshold switching for pH and glucose. (c) Top view of a patch after insertion into porcine skin: bright‐field image (1), corresponding fluorescence (2), and converted QR code (3). (d) Time‐resolved QR code output under physiological conditions (pH 7.4, glucose 5 mM) showing stabilization after 30 min and accurate readout for ∼1 h. (e) Representative QR codes generated from patches exposed to all pH/glucose combinations (*n* = 27 patches). (f,g) Confusion matrices for pH and glucose classification across nine patches per condition (*n* = 27 patches). (h) Quantitative analysis confirming robust and reproducible performance, with classification accuracies of 93% for pH and 85% for glucose (*n* = 27 patches). Green fluorescence indicates sensing microneedles, while red fluorescence marks reference microneedles for orientation and insertion confirmation. Scale bars: 1 mm.

We next fabricated a 5 × 5 QR code microneedle patch for multianalyte detection of pH and glucose by co‐patterning FL and FLB probes within the same array (Figure 5a,b). Each threshold was represented by three microneedles distributed across the array to minimize localized variability and enable triplicate measurements. In total, nine needles were dedicated to pH sensing, nine to glucose detection, and seven doped with Nile Red served as reference markers for QR code orientation and signal consistency (Figure 5b).

To evaluate the stability and accuracy under physiological conditions, we monitored patch performance following insertion in porcine skin incubated in interstitial fluid (ISF) at pH 7.4 and 5 mM glucose (Figure 5c,d and Figure S8). Fluorescence images were acquired between 5 min and 12 h postinsertion, and corresponding QR codes were generated as two‐color digital outputs. A majority‐vote classification across replicate needles assigned pH and glucose ranges, reported as central values with associated uncertainties of ±0.5 pH units and ±1 mM glucose (Figure S10a,b). The results revealed a 30 min settling time, during which the QR code pattern evolved before stabilizing (Figure S8), consistent with the equilibration kinetics of FL and FLB probes in the PVA shell (Figure S5). Once stabilized, the QR code remained stable and accurately decoded for ∼3 h, corresponding to an effective operational window of 2.5 h after equilibration. Beyond this period, degradation of the PVA matrix compromised structural integrity and signal fidelity, while the PLGA core dissolved completely within 14 days [25]. Within the operating window, the digital output is reversible: alternating the surrounding analyte conditions induces reproducible QR‐code switching with a characteristic stabilization time of ∼30 min, consistent with diffusion/equilibration through the hydrated PVA shell, and the resulting patterns are robustly decoded by the algorithm (Figure S8).

We next validated the performance of the QR code microneedle sensor for simultaneous pH and glucose detection in porcine skin incubated with ISF solutions containing various combinations of analyte concentrations. The readout was performed 30 min after insertion to ensure signal stabilization. Figure 5e shows representative QR codes generated by the patches, illustrating dynamic fluorescence patterns in response to analyte changes (Figure S9e shows similar results for synthetic skin incubated in PBS). To quantify reliability, Figure 5f,g present confusion matrices for pH and glucose, generated from nine independent measurements per condition across 27 patches. The sensor achieved 93% accuracy for pH detection and 85% accuracy for glucose (Figure 5h), with comparable F1 scores confirming balanced performance. Notably, robust digital classification is preserved in this complex biological environment, i.e., ISF‐incubated porcine skin, which inherently contains proteins, salts, lipids/fatty acids, and other biomolecules that could act as potential interferents.

We further evaluated reproducibility across independent microneedle patches by analyzing detection rates and consistency under physiological conditions of pH 7.4 and 5 mM glucose (Figure S10c,d). pH probes achieved 100% precision and 90% accuracy, demonstrating highly reproducible identification of the target pH. Glucose probes showed slightly greater variability but still performed well, with 88% precision and 70% accuracy, reliably detecting 5 mM glucose in most cases. Importantly, the QR code patch design—featuring triplicate probes for threshold—mitigates local errors such as incomplete insertion or tip detachment. This built‐in redundancy enhances reliability by enabling majority‐rule classification and compensating for outliers. Together, these results confirm that the QR code microneedle patch provides robust, reproducible digital readouts for both pH and glucose under physiologically relevant conditions. While occasional needle‐level failures may occur, the modular, fault‐tolerant architecture ensures consistent performance, establishing the platform as a promising tool for real‐time biosensing and digital health monitoring.

## 3 Conclusions

This work introduces a digital paradigm for transdermal sensing through fluorescent microneedles that encode biochemical information into scannable QR code patterns. By engineering sharp, threshold‐based fluorescence activation, we move beyond analog intensity quantification and mitigate key limitations of conventional fluorescent microneedle readouts, including calibration dependence, drift, and susceptibility to tissue‐and application‐dependent variability. Using a high‐contrast binary (“on/off”) decision at each microneedle, the platform enables robust, multiplexed sensing, demonstrated here for pH and glucose, with a robust QR‐code binary digital output compatible with portable, low‐power optical readers and consumer‐grade imaging devices.

Within this digital sensing framework, the effective measurement resolution is defined by the spacing and number of encoded thresholds rather than by incremental analog intensity sensitivity. In the present proof‐of‐concept, threshold spacing of 1 pH unit and 2 mM glucose corresponds to intrinsic quantization uncertainties of ±0.5 pH and ±1 mM, respectively. This trade‐off is intentional: it prioritizes calibration‐free robustness and fault tolerance in transdermal environments where optical heterogeneity, insertion depth, illumination geometry, and time‐dependent effects can obscure small analog intensity changes. At the same time, the modular architecture supports rapid expansion to additional analytes by incorporating orthogonal probes and offers clear routes to increase resolution while preserving digital robustness, including expanding threshold density/array size and implementing multichannel or ratiometric thresholding to further suppress optical variability and reduce the impact of occasional needle‐level outliers.

Looking ahead, priorities include extending the operational time window through optimized matrix formulations and degradation kinetics, sharpening probe switching behavior through probe/matrix engineering, and integrating automated or wireless readout workflows for user‐independent decoding. For example, microneedle fluorescence could be captured with a smartphone camera coupled to a small reusable clip‐on module providing excitation/emission filtering, followed by app‐based automated segmentation and QR‐pattern decoding. Translation to in vivo models will be essential to validate performance under complex biological conditions and to refine biocompatibility, deployment dynamics, and resorption profiles. With these advances, digital microneedles could evolve into a scalable interface for calibration‐free biochemical monitoring, enabling clinically actionable, decentralized diagnostics and data‐driven personalized healthcare.

## 4 Experimental Section

### 4.1 Ethical Compliance

Porcine skin samples were obtained from animals euthanized for independent experimental purposes at the University of Pisa. As no animals were sacrificed specifically for this study and tissues were reused in accordance with the 3Rs principles (Replacement, Reduction, and Refinement), ethical approval was not required under applicable institutional and national regulations.

### 4.2 Statistical Analysis

All results are presented as mean ± standard deviation (SD), with a minimum of three independent replicates (*n* ≥ 3), as specified in the corresponding figure captions. Statistical analysis of cellular experiments was performed using one‐way analysis of variance (ANOVA) followed by the Newman‐Keuls multiple comparison test. Differences were considered statistically significant at *p* < 0.05. Data processing and statistical analyses were performed using MATLAB and Python.

## Author Contributions

G.B. conceived and supervised the project. F.A. designed and fabricated the microneedle devices and performed the optical, mechanical, and sensing characterization. F.A. and S.V. developed the software for QR‐code readout and interpretation. E.B., P.O., and G.B. carried out the in vitro cytotoxicity and biosafety studies. M.C. and F.A. performed the OCT measurements and data analysis. I.N. supported F.A. in experiments with porcine skin. G.B., F.A., S.V., E.B., M.C., P.O., G.B., and I.N. analyzed the data and prepared the figures. G.B. and F.A. wrote the manuscript with input from all authors. All authors discussed the results and approved the final manuscript.

## Conflicts of Interest

The authors declare no conflicts of interest.

## Supporting information




**Supporting File**: adma72701‐sup‐0001‐SuppMat.pdf.

## Data Availability

The data that support the findings of this study are available from the corresponding author upon reasonable request.
