# Supplementary material for: Digital Microneedles for Multiplexed Transdermal Sensing via Fluorescent QR Codes
Source: Adv Mater. 2026 Mar 10;38(36):e18935. doi: 10.1002/adma.202518935 (PMC13310096; doi:10.1002/adma.202518935)
Supplement: Supplementary file 1 — Supporting File: adma72701‐sup‐0001‐SuppMat.pdf. [file ADMA-38-e18935-s001.pdf]

Supporting Information

Digital Microneedles for Multiplexed Transdermal Sensing via Fluorescent QR Codes

Farbod Abazar[1], Shahrokh Vahabi[1], Elena Bellotti[1], Martina Corsi[1], Irene Nocera[2], Paola Orlandi[3], Guido Bocci[4], Giuseppe Barillaro[1*]

[1] Information Engineering Department, University of Pisa, via G. Caruso 16, 56122 Pisa, Italy.

[2] Department of Veterinary Sciences, University of Pisa, 56122 Pisa, Italy.

[3] Department of Clinical and Experimental Medicine, School of Medicine, University of Pisa, 56126 Pisa, Italy

[4] Department of Translational Research and New Technologies in Medicine and Surgery, School of Medicine, University of Pisa, 56126 Pisa, Italy

[*]Corresponding author: giuseppe.barillaro@unipi.it

Experimental Section

Materials and chemicals

Nile red (technical grade), fluorescein sodium salt, polyvinyl alcohol (PVA, 80% hydrolyzed, Mw = 9,000-10,000), polyvinyl alcohol (PVA, 98% hydrolyzed, Mw = 31,000-50,000), sucrose (≥99.5%), D-(+)-Glucose (ACS reagent), sodium hydroxide (NaOH, 98%), aqueous hydrochloric acid (HCl, 37%), poly(D,L-lactide-co-glycolide) (PLGA, 85:15 lactide:glycolide, Mw = 50,000-75,000), sodium phosphate monobasic monohydrate (PBS, 98%), sodium chloride (NaCl, 99%), Magnesium sulfate ($MgSO_4$, 98%), potassium chloride (KCl, 99%), HSA, α-globulins Cohn (fraction IV-4) human, penicillin, streptomycin, heat-inactivated fetal bovine serum (FBS), L-glutamine, heparin, type A gelatin were purchased from Sigma Aldrich (Germany). Fluorescein boronic acid (96%) was purchased from abcr GmbH (Germany). Absolute ethanol (EtOH, 99.9%) was purchased from Carlo Erba Reagents

(Italy). 1,4-dioxane (≥99% stabilized with 25 ppm BHT) was purchased from VWR (USA). PDMS (polydimethylsiloxane, Sylgard 184) base and thermal curing agent were purchased from Cecchi S.r.l. (Italy). Synthetic human tissues (2N) were purchased from SynDaver (USA). All buffers were prepared in deionized water (DIW) and pH adjusted with NaOH (5 M) and HCl (1 M) aqueous solution. Aqueous solutions were prepared using deionized water (DIW, 15 MΩ×cm) filtered by Elix®, Merck Millipore (Germany). Porcine skin samples were obtained at the University of Pisa from animals euthanized for independent experimental purposes, in strict adherence to the 3Rs principles (Replacement, Reduction, and Refinement), whereby animal use was minimized by reusing tissues whenever possible. Abdominal flank skin was collected from adult female Göttingen minipigs and stored in sterile 0.9% (w/v) NaCl solution until use. Recombinant human epidermal growth factor (rhEGF), and recombinant human basic fibroblast growth factor (rhbFGF), were purchased from PrepoTech EC Ltd (London). Normal human dermal fibroblast (HNDF) cells were purchased from ATCC (Manassas, VA, USA). MCDB-131 culture medium was purchased from Gibco (Gaithersburg, MD, USA).

Design and fabrication of QR code microneedle (MN) patch

The microneedle (MN) patch was fabricated using a conventional molding technique based on a polydimethylsiloxane (PDMS) negative mold. A high-resolution master mold was first produced from a 3D STL design file using a microArch S230 3D printer (BMF Material Technology Inc., Shenzhen, China) with HTL yellow resin. The design featured a 5×5 array of microneedles with a center-to-center spacing of 1600 μm arranged within an 8 mm × 8 mm footprint (Figure S1a-c). Each microneedle had a conical geometry with a base radius of 160 μm, a height of 800 μm, and a tip radius of approximately 5 μm (Figures 2h, S1c). To facilitate tip detachment and enable spatial encoding, the patch design also incorporated pedestal structures located at the base of each microneedle. These pedestals had a base

diameter of 1500 μm, a top diameter of 160 μm, and a height of 880 μm, serving both to elevate the needles above the patch backing and to act as selective filling channels for the needle tips. To fabricate the PDMS mold, the base and curing agent were mixed in a 10:1 weight ratio for 5 minutes. The mixture was then degassed under vacuum for 30 minutes to remove trapped air bubbles. After degassing, it was poured over the 3D-printed master mold and cured at 60 °C for 5 hours. This process yielded a PDMS negative mold with precisely replicated inverted microneedle and pedestal structures.

A core–shell microneedle architecture was employed to fabricate patches functionalized with pH- and glucose-responsive fluorescent probes. The shell was composed of poly(vinyl alcohol) (PVA), chosen for its hydrophilicity and biocompatibility. To prepare the shell solution, 2 g of PVA (Mw = 31,000–50,000) was dissolved in 20 mL of deionized (DI) water by microwave heating for 15 seconds. The solution was then allowed to cool to room temperature under a fume hood for 30 minutes, then either fluorescein (FL) or fluorescein boronic acid (FLB) at different concentrations were added to the PVA solution. FL was added at 0.001%, 0.006%, and 0.0085% (w/v) for pH sensing, while FLB was added at 0.003%, 0.015%, and 0.025% (w/v) for glucose sensing. Each concentration was probed in triplicate within the patch. Each solution was stirred for 30 minutes to ensure homogeneity. Approximately 5 μL of each shell solution was selectively dispensed into the desired microneedle cavities of the PDMS mold based on the predefined QR code layout. The mold was centrifuged at 3000 rpm for 20 minutes to ensure uniform filling and was then held at 40 °C for 30 minutes to initiate drying. To remove any residual casting solution and promote clean shell formation, a 1:1 (v/v) mixture of DI water and ethanol was prepared. About 5 μL of this washing solution was drop-cast onto each microneedle cavity and centrifuged for 20 minutes. This washing step was repeated at least three times to fully cleanse and fill the mold cavities. After shell formation, the mold was placed in an oven at 60 °C for 12 hours to fully dry and solidify the PVA shell, completing the formation of the probe-loaded shell structure. For core

filling, a 5% (w/v) solution of poly(lactic-co-glycolic acid) (PLGA) was prepared by dissolving PLGA in a solvent mixture of dioxane and deionized (DI) water in a 95:5 (v/v) ratio.

For reference microneedles, Nile Red was added to a separate PLGA solution at a concentration of 0.025% (w/v). Approximately 5 µL of the desired core solution was selectively drop-cast into the appropriate microneedle cavities of the mold. The mold was then centrifuged at 3000 rpm for 20 minutes and incubated at 40 °C for 30 minutes to promote uniform filling and initial drying. To remove any excess core solution and ensure precise cavity formation, approximately 5 µL of pure dioxane was drop-cast onto each cavity and centrifuged again for 20 minutes. This washing process was repeated at least three times to ensure full removal of residual solution and consistent cavity filling. Finally, the mold was placed in an oven at 60 °C for 12 hours to fully dry the core material and complete the fabrication of the microneedle structure.

As the final fabrication step, the needle pedestal and patch backing was formed by casting a solution composed of 18% (w/v) PVA (Mw = 9,000–10,000) and 18% (w/v) sucrose dissolved in deionized (DI) water. Approximately 200 µL of this solution was applied to the top surface of the dried PDMS mold containing the microneedle structures. Due to the difference in surface tension between the PDMS mold and the aqueous backing solution, a trapped air bubble formed between each microneedle and its underlying pedestal. This air gap is a critical feature that facilitates controlled tip detachment upon application. The mold was left under a chemical hood for 6 hours to allow partial drying, followed by placement in an oven at 40 °C for 24 hours to ensure complete solidification of the backing layer. After drying, the fully formed microneedle patch was carefully peeled from the mold (Figure 2d, S1d–f).

The demolded microneedle patches were stored in a desiccator until further use to preserve structural integrity and prevent moisture uptake.

Mechanical characterization of the MN patch

The mechanical properties of QR code microneedle (MN) patches—with and without embedded air bubble structures—were evaluated by characterizing their compression and shear stress-strain behavior using a custom-built, LabVIEW-controlled test setup (Figure S3a). The system included a motorized translation stage (Sigma Koki, SGSP80-20ZF; travel per pulse: 0.2 µm; max travel: 20 mm; max speed: 2 mm/s) equipped with a 20 N load cell (Omega LCMFD-20N; sensitivity: 2.20 mV/V; accuracy: 0.03 N), and a SourceMeter unit used for load cell biasing and readout (Keithley SMU 2410). Precise motion control was achieved using a 2-axis stage controller (Sigma Koki SHOT-302GS; min speed: 1 pulse/s; max speed: 500,000 pulses/s).

To assess compression strength measurements, a microneedle patch was mounted on top of the translation stage with the microneedles oriented upward (Figure S3b). The translation stage was programmed to move upward at speeds of 0.5 mm/min and 4 mm/min, compressing the MNs vertically against a fixed stage. The initial distance between the MN tips and the stage was set to 1 cm. Upon first contact between the MN tips and the fixed stage, the displacement was zeroed, and force measurements were recorded against displacement as the sensor continued its upward motion. Compression force was measured until a displacement of 0.4 mm was reached from the needle tip toward the patch backing.

To evaluate the skin insertion force, the same procedure was applied using porcine skin (5 cm × 5 cm), which was stretched and affixed to the top of the translation stage, while the microneedle patch was mounted upside down on the fixed stage (Figure S3c).

For shear strength measurements, a microneedle patch was vertically mounted onto a stainless-steel cube using adhesive glue. This assembly was fixed to the top of the translation stage, while a second stainless-steel cube was attached to the fixed stage and aligned to overlap the microneedles by approximately 1 mm in the horizontal direction and positioned about 1 mm above the top row of needles in the vertical

direction (Figure S3d). The translation stage advanced at speeds of 0.5 mm/min and 4 mm/min. Displacement and force measurements were initiated upon the initial contact between the microneedles and the opposing cube, and data collection continued as the stage traveled 1 mm in the vertical direction.

Ex vivo skin insertion of the MN patch

To evaluate the penetration and detachment efficiency of the microneedles, patches with microneedle tips fully loaded with Nile Red were applied to porcine skin in an *ex vivo* setup. Full-thickness porcine skin samples (5 cm × 5 cm) were stretched and secured to a flat surface using four screws to ensure tension and stability during application. Each patch was manually pressed onto the skin using a thumb for 30 seconds.

To assess penetration efficiency alone, microneedle patches were applied using vertical force only and immediately removed without applying any shear stress. A blue tissue-marking dye (Mark-It™, Richard-Allan Scientific) was then applied to the skin surface for 5 minutes to stain the microneedle insertion sites. Residual dye was removed with alcohol pads prior to imaging.

To assess tip detachment efficiency, after penetration a gentle sliding motion was then applied in one direction along the skin surface, generating shear force between the microneedles and the backing layer. After removing the patch backing, the skin surface was examined under an optical microscope (DM2500 M, Leica Microsystems) equipped with an I3 fluorescence filter cube (excitation: BP 450–490 nm; emission: LP 515 nm) to identify any microneedle tips embedded within the tissue.

Penetration and detachment efficiencies were quantified by counting either the number of stained puncture sites (dye-based) or fluorescent microneedles retained in the skin (Nile Red-based), and normalizing to the total number of microneedles in each patch (n = 25 per patch).

Kinetics evaluation of FL and FLB probes for glucose and pH sensing

To study the time-resolved fluorescence response of the sensing probes, MN patches composed of a 5 × 5 array were prepared containing either fluorescein (FL) or fluorescein boronic acid (FLB). Each microneedle featured a core–shell architecture, with the shell made of poly(vinyl alcohol) (PVA) doped with either FL for pH sensing or FLB for glucose sensing, and the core composed of PLGA. Each needle in the array was loaded with the same probe concentration to evaluate fluorescence intensity kinetics and reproducibility across replicates.

Synthetic skin samples (SynDaver; 3 cm × 3 cm, 1.5 mm thick) were preconditioned by soaking overnight in 40 mL of phosphate-buffered saline (PBS; 10 mM PBS with 100 mM NaCl) at pH 5.5, 7.4, or 8.5 (without glucose), or at pH 7.4 with glucose concentrations of 1, 5, or 10 mM. MN patches were inserted and deployed into the synthetic skin as described in the *Ex vivo skin insertion of the MN patch* section. Fluorescence images were captured from the top surface of the skin at time points of 0, 5, 15, 30, 60, and 180 minutes using an optical microscope (DM2500 M, Leica Microsystems) with an L5 fluorescence filter cube (excitation: BP 480–440 nm; emission: BP 527–530 nm). Multiple overlapping images were acquired in dark-field mode using a 2.5× objective lens focused on the top surface of the patch, then stitched together to form a complete view of the microneedle array. Samples were kept in sealed petri dishes during imaging to maintain consistent hydration (Figure S5a). Fluorescence intensity at each time point was quantified by isolating the green channel from each image and calculating the average Value (V) from the HSV color model (0–255 scale) using a custom image-processing script (Figure S5b,c).

Threshold Calibration for Digital Fluorescence Readout

For digital threshold calibration, 5×5 microneedle patches with core–shell architecture were fabricated as described above. For a given patch, each column of five microneedles was loaded with the same concentration of either FL or FLB, allowing the evaluation of probe-specific activation thresholds and reproducibility. Different columns were doped with varying fluorophore concentrations to span a range of sensitivity thresholds. FL was incorporated into the PVA shell at concentrations of 0.001%, 0.003%, 0.006%, 0.008%, and 0.0085% (w/v) for pH sensing, while FLB was added at 0.003%, 0.008%, 0.015%, 0.020%, and 0.025% (w/v) for glucose detection. The patches were inserted into synthetic skin samples preconditioned at pH values ranging from 4.5 to 8.5 in 1-unit increments or glucose concentrations from 1 to 10 mM in 2 mM steps, and incubated for 24 hours. Fluorescence images were acquired 30 minutes after insertion using an optical microscope (DM2500 M, Leica Microsystems) equipped with an L5 fluorescence filter cube (excitation: BP 480–440 nm; emission: BP 527–530 nm). Multiple overlapping images were acquired in dark-field mode using a 2.5× objective lens focused on the top surface of the patch, then stitched together to form a complete view of the microneedle array. Fluorescence intensity was quantified by isolating the green channel from each image and calculating the average Value (V) from the HSV color space (scale: 0–255) for each column in the patch using a custom image-processing script.

Digitalization of fluorescence MN images

Fluorescence imaging of the QR-code MN patches was performed under three conditions: (i) as-fabricated patches in air, (ii) microneedles inserted into synthetic skin (*in vitro*), and (iii) microneedles inserted into porcine skin (*ex vivo*).

To convert these fluorescence images into digital images, a custom Python-based image processing pipeline was developed using OpenCV. The workflow began with capturing fluorescence images of

microneedles inserted into skin (Figure S6a,b) and subsequently converting the image into HSV (Hue, Saturation, Value) color space. This transformation enabled more robust and consistent detection of fluorescence signals corresponding to specific fluorophores—red for Nile Red and green for FL/FLB. Color-specific HSV thresholds were defined for digital classification as follows: Red (Nile Red): H = [0–10] ∪ [170–180], S ≥ 120, V ≥ 70; Green (FL): H = [45–85], S ≥ 50, V ≥ 50; Green (FLB): H = [45–85], S ≥ 50, V ≥ 30. These ranges were empirically determined to capture the characteristic fluorescence profiles of each probe under the specified imaging conditions. Among the HSV parameters, the Value (V) component—representing image brightness—was used as the primary criterion for fluorescence thresholding. This allowed the algorithm to distinguish between an "on" signal (fluorescent activation) and background noise based on intensity, independent of slight variations in hue or saturation that might arise due to imaging conditions or probe diffusion. By applying V-based thresholds, the classification system robustly identified whether each microneedle was activated, thereby enabling binary encoding of probe response.

Binary masks were generated for each channel to isolate color-positive regions. Morphological operations (e.g., opening and dilation), implemented via the OpenCV function "cv2.morphologyEx", were applied to reduce noise and enhance the integrity of detected fluorescence regions. These steps ensured accurate probe identification by minimizing background artifacts and enabling reliable on/off classification. Contours of the isolated fluorescence regions were extracted using the OpenCV function "cv2.findContours", which also enabled robust detection of array position and alignment—even under conditions of slight distortion or rotation. Each fluorescence image was segmented into a 5×5 matrix, with each cell corresponding to an individual microneedle in the array (Figure S6c). For each grid cell, the presence or absence of a fluorescence signal was assessed based on the defined HSV thresholds. Digital values were assigned as follows: 0 = no signal; 1 = green fluorescence (FL or FLB); 2 = red fluorescence (Nile Red). This resulted in a 25-element digital matrix representing the fluorescence status

of each microneedle (Figure S6d-f). The final matrix served as a machine-readable QR code that encoded the biochemical response profile (e.g., pH or glucose level).

In QR code MN patches designed for multianalyte sensing of pH and glucose, each concentration threshold was represented by three microneedles within the array—i.e., three needles were loaded with the same concentration of either FL or FLB. This triplicate configuration allowed internal replication for each sensing condition. To assign the corresponding pH or glucose range encoded in the QR code, a classification algorithm embedded within the image-processing script applied a majority-vote rule across the three replicate needles for each concentration threshold. This approach enhanced the robustness of the digital readout by minimizing the influence of outlier signals or local variability.

Assessment of the operational time window of the QR code glucose/pH microneedle patch

To determine the optimal operational time window of QR code microneedle patch for simultaneous sensing of glucose and pH, the performance consistency across five independent patches was evaluated.

Synthetic skin samples (SynDaver; 3 cm × 3 cm × 1.5 mm) were preconditioned overnight in 40 mL of phosphate-buffered saline (PBS; 10 mM PBS with 100 mM NaCl) at pH 7.4 containing 5 mM glucose, simulating physiological conditions. The QR code MN patches were inserted and deployed into the preconditioned synthetic skin samples as described in the *Ex Vivo Skin Insertion of the MN patch* section. The skin samples containing the microneedles were then placed inside a custom-designed chamber (Figure S8a), which featured a liquid reservoir at the bottom and a mounting system that secured the skin sample in contact with the liquid using an O-ring. The entire chamber was enclosed in a sealed Petri dish and incubated at 37 °C to mimic physiological temperature conditions. Fluorescence images were acquired from the top surface of the skin at multiple time points: 5, 15, 30, 60, 180, 360, and 720 minutes. Image analysis was conducted by converting the fluorescence images into digital QR codes representing

microneedle activation states. These binary outputs were then compared against the expected QR code response to assess degradation over time (Figure S8b).

Assessment of the cytocompatibility of the microneedles

Normal human dermal fibroblast (HNDF) cells were used to evaluate the cytocompatibility of the microneedle (MN) extracts. Cells were maintained in MCDB-131 culture medium supplemented with 100 U/mL penicillin, 100 μg/ml streptomycin, 20% heat-inactivated fetal bovine serum (FBS), 2 mM L-glutamine, 10 IU/mL heparin, 10 ng/mL recombinant human epidermal growth factor (rhEGF), and 5 ng/mL recombinant human basic fibroblast growth factor. Cells were cultured in tissue culture flasks coated with type A gelatin and maintained at 37°C in a humidified atmosphere containing 5% CO_2. MN were incubated in MCDB-131 medium at a concentration of 100 μg/mL for 24 or 48 h to obtain conditioned extracts. Prior to cell treatment, the MN-conditioned media were sterile-filtered using a 0.2 μm syringe filter and serially diluted with fresh culture medium to obtain final concentrations of 1 μg/mL, 100 ng/mL, 10 ng/mL, and 1 ng/mL. For cytocompatibility experiments, HDNF cells were seeded in 24-well plates at a density of 1 x 10^5 cells per well and allowed to adhere for 24 h. Subsequently, the culture medium was replaced with MN-conditioned medium at different concentration obtained after 24 and 48 h. Untreated cells cultured in fresh medium were used as control. All the experiments were performed in triplicate. After 24 h of exposure, cells were harvested with trypsin/EDTA and the viable ones manually counted using a hemocytometer. Cell proliferation was evaluated by comparing the number of cells treated with the MN-conditioned medium with those of the control group (Figure 3s, S4).

Preparation of synthetic ISF

Synthetic interstitial fluid (ISF) solutions were prepared to mimic the ionic, metabolic, and protein composition of physiological ISF. The synthetic ISF consisted of NaCl (107.7 mM), KCl (3.48 mM), $CaCl_2$ (1.53 mM), $MgSO_4$ (0.69 mM), $NaHCO_3$ (26.2 mM), NaH_2PO_4 (1.67 mM), and glucose (1, 5, or 10 mM), with the pH adjusted to 5.5, 7.4, or 8.5. To account for the protein content of ISF, the solution was supplemented with a total protein concentration of 11 g L^{-1}, consisting of human serum albumin (HSA, 1 μM) and α-globulins at a 60:40 ratio.

Assessment of the reversibility of the QR code glucose/pH microneedle sensor response

The reversibility of the QR code microneedle (MN) patch for simultaneous glucose and pH sensing was evaluated using cyclic changes in solution composition while maintaining the same skin sample and chamber configuration throughout the experiment. Five independent QR code MN patches were tested to assess response reproducibility. Synthetic skin samples (SynDaver; 3 cm × 3 cm × 1.5 mm) were first equilibrated overnight in phosphate-buffered saline (PBS; 10 mM PBS with 100 mM NaCl) adjusted to pH 5.5 and containing 1 mM glucose. The same solution was also loaded into the liquid reservoir of the custom-designed chamber. Following equilibration, the QR code MN patches were inserted into the synthetic skin and mounted in the chamber, which was sealed using an O-ring and enclosed in a Petri dish. The entire assembly was maintained at 37 °C to mimic physiological conditions. After microneedle insertion, the system was allowed to equilibrate for 30 minutes, after which fluorescence images were acquired from the top surface of the skin. Subsequently, the solution in the chamber was exchanged with PBS (10 mM PBS with 100 mM NaCl) adjusted to pH 8.5 and containing 10 mM glucose, while keeping the MN patch and skin sample in place. After an additional 30-minute equilibration period, fluorescence images were collected again. This process of alternating between acidic/low-glucose (pH 5.5, 1 mM glucose) and basic/high-glucose (pH 8.5, 10 mM glucose) solutions was repeated for multiple cycles to

evaluate sensor reversibility. Fluorescence images obtained at each cycle were converted into digital QR codes representing the activation states of the pH- and glucose-responsive microneedles. The decoded QR outputs were compared with the expected responses for each solution condition (Figure S8d).

Assessment of the sensing performance of the QR-code Glucose/pH microneedle patch

- In vitro (Synthetic skin model)

To evaluate the sensing performance of the QR-code Glucose/pH MN patch under physiopathologically relevant conditions, including variations in both pH and glucose levels, synthetic skin samples (SynDaver; 3 cm × 3 cm × 1.5 mm) were soaked overnight in 40 mL of phosphate-buffered saline (PBS; 10 mM PBS with 100 mM NaCl) containing distinct combinations of pH and glucose: pH 5.5, 7.4, and 8.5, each combined with 1 mM, 5 mM, and 10 mM glucose. This resulted in a total of nine test conditions covering the range of clinically relevant pH and glucose levels.

Microneedle patches were inserted into the prepared synthetic skin samples following the procedure described in the *Ex Vivo Skin Insertion of the MN patch* section. The skin samples containing the microneedles were then sealed in Petri dishes and incubated for 30 minutes to allow equilibration. Fluorescence images were subsequently acquired from the top surface of the skin. To assess patch performance, the captured images were processed using the custom image-processing script to generate digital QR codes, which were then converted into binary matrices and compared with the predicted responses. Each measurement condition was tested with at least three independent patches, and each test was repeated three times to evaluate accuracy and reproducibility. To further assess the reliability of the patch under stable conditions, the same procedure was repeated using synthetic skin incubated at fixed physiological conditions (pH 7.4, 5 mM glucose) as a control (Figure S10).

- *Ex vivo (Cadaver porcine skin model)*

To evaluate the sensing performance and selectivity of the QR-code Glucose/pH microneedle (MN) patch in an *ex vivo* biological tissue model, experiments were conducted using cadaver porcine skin under physiopathologically relevant pH and glucose conditions. Excised porcine skin samples were first incubated overnight in phosphate-buffered saline (PBS; 10 mM PBS with 100 mM NaCl) at pH 7.4 without glucose to remove residual metabolites and equilibrate the tissue. Following this initial equilibration, the PBS solution was removed and replaced with synthetic interstitial fluid (ISF) containing defined pH and glucose concentrations. The ISF composition matched that used in the synthetic skin experiments supplemented with glucose (1, 5, or 10 mM) and adjusted to pH 5.5, 7.4, or 8.5. The porcine skin samples were incubated overnight in the target ISF solution to allow diffusion and equilibration of glucose and pH within the tissue matrix.

QR-code MN patches were then inserted into the prepared porcine skin samples following the same *in vivo* insertion procedure described previously for synthetic skin. After insertion, the skin samples containing the microneedles were sealed in Petri dishes and incubated for 30 minutes to allow sensor equilibration. Fluorescence images were subsequently acquired from the top surface of the skin. The captured images were processed using the custom image-processing script to generate digital QR codes, which were converted into binary matrices and compared with the predicted responses corresponding to the applied pH and glucose conditions. Each experimental condition was tested using at least three independent MN patches, and measurements were repeated three times to assess sensing accuracy, reproducibility, and selectivity in the *ex vivo* porcine skin model.

Statistical Analysis

All results are presented as mean ± standard deviation (SD), with a minimum of three independent replicates ($n \geq 3$). Statistical analysis of cellular experiments was performed using one-way analysis of variance (ANOVA) followed by the Newman-Keuls multiple comparison test. Differences were considered statistically significant at $p < 0.05$. Data processing and statistical analyses were performed using MATLAB and Python.

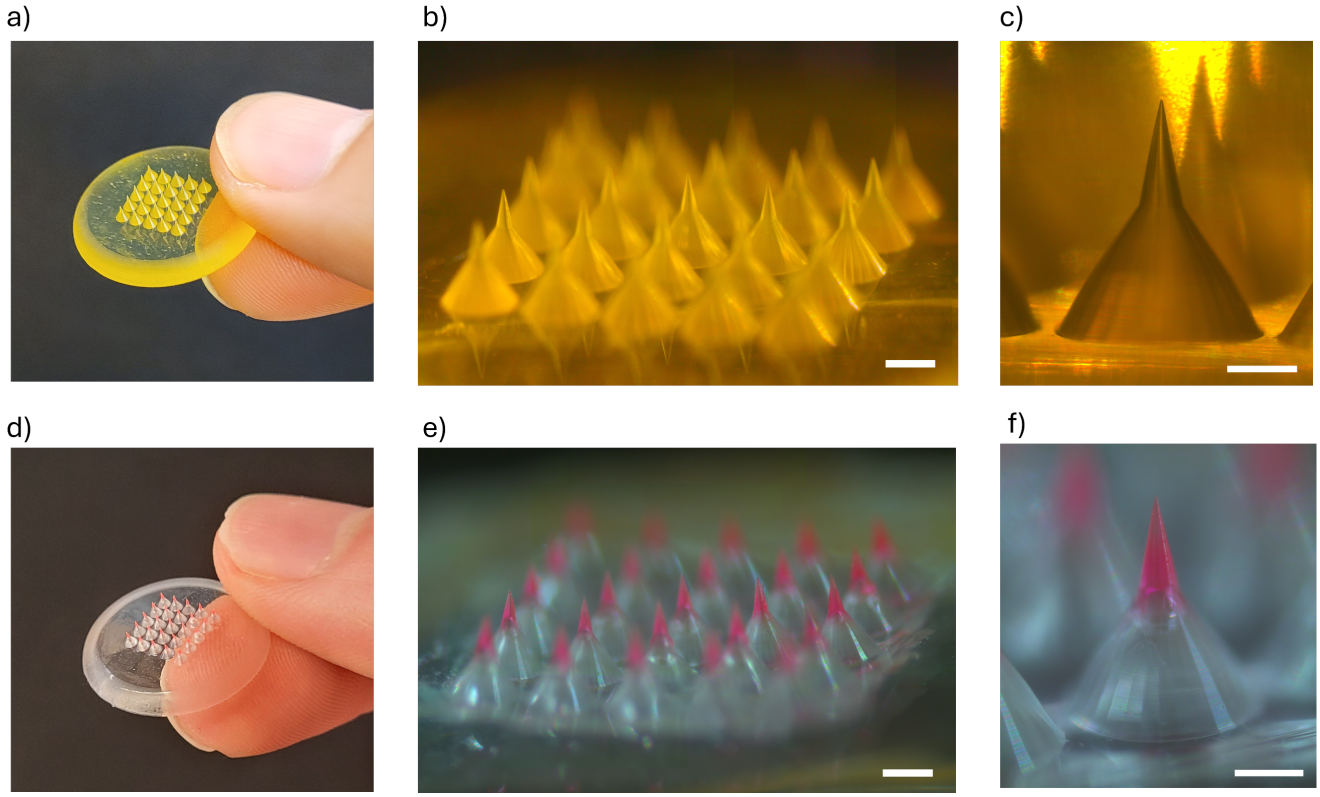

Figure S1. Comparison of 3D-printed and replica-molded microneedle patches. (a) Photograph of a 3D-printed microneedle array with high resolution digital light processing DLP method, accompanied by (b) a bird's-eye view (scalebar 1 mm), and (c) a close-up view of a single microneedle (scalebars 500 µm). (d) Photograph of a replica-molded microneedle patch, with (e) a corresponding bird's-eye view (scale bar 1 mm) and, (f) an image of a single microneedle (scale bars 500 µm).

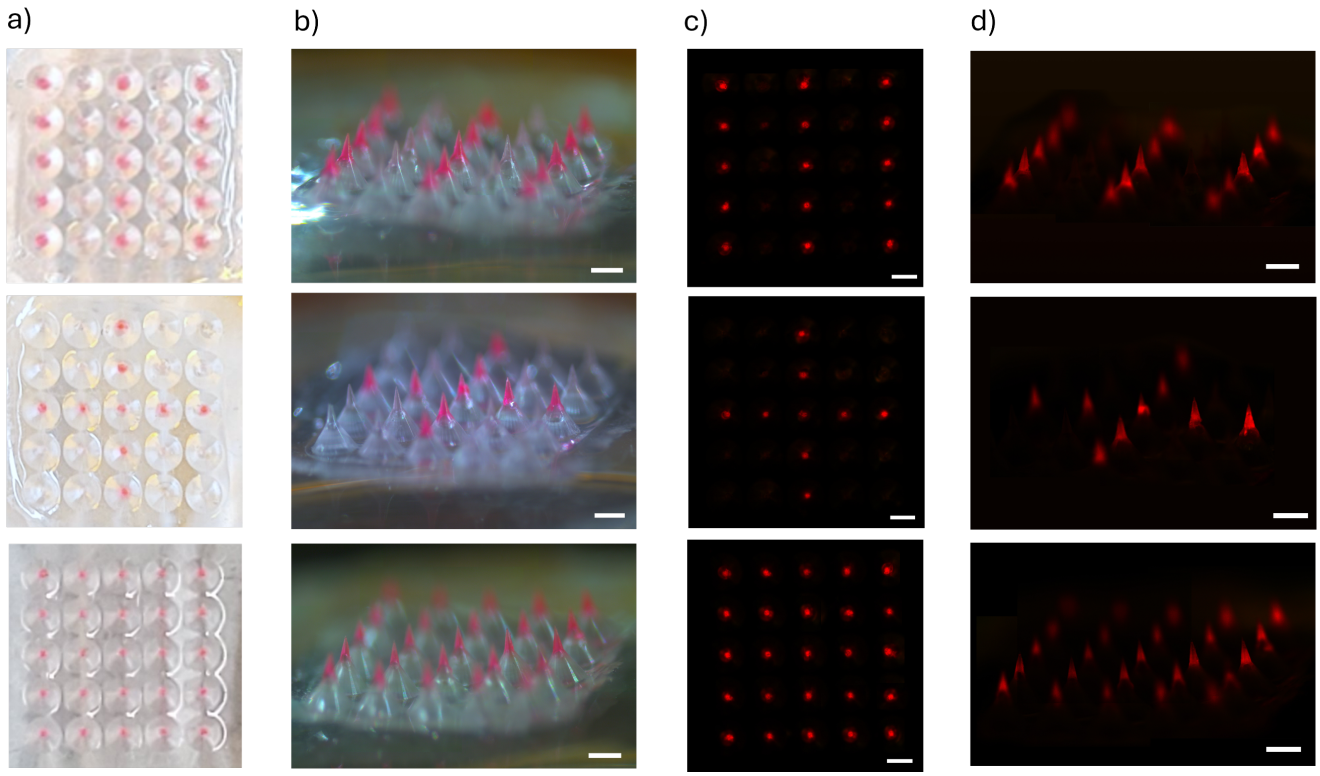

Figure S2. Demonstration of patterning flexibility for fluorescent microneedle patches. a,b) Bright-field top view (a) and bird's-eye (b) images of microneedle patches loaded with Nile Red demonstrating different spatial patterns. c,d) Corresponding fluorescence images in top-view (c) and bird's-eye (d) modes, demonstrating the ability to fabricate customizable microneedle patches with versatile spatial patterns. Scale bars: 1 mm.

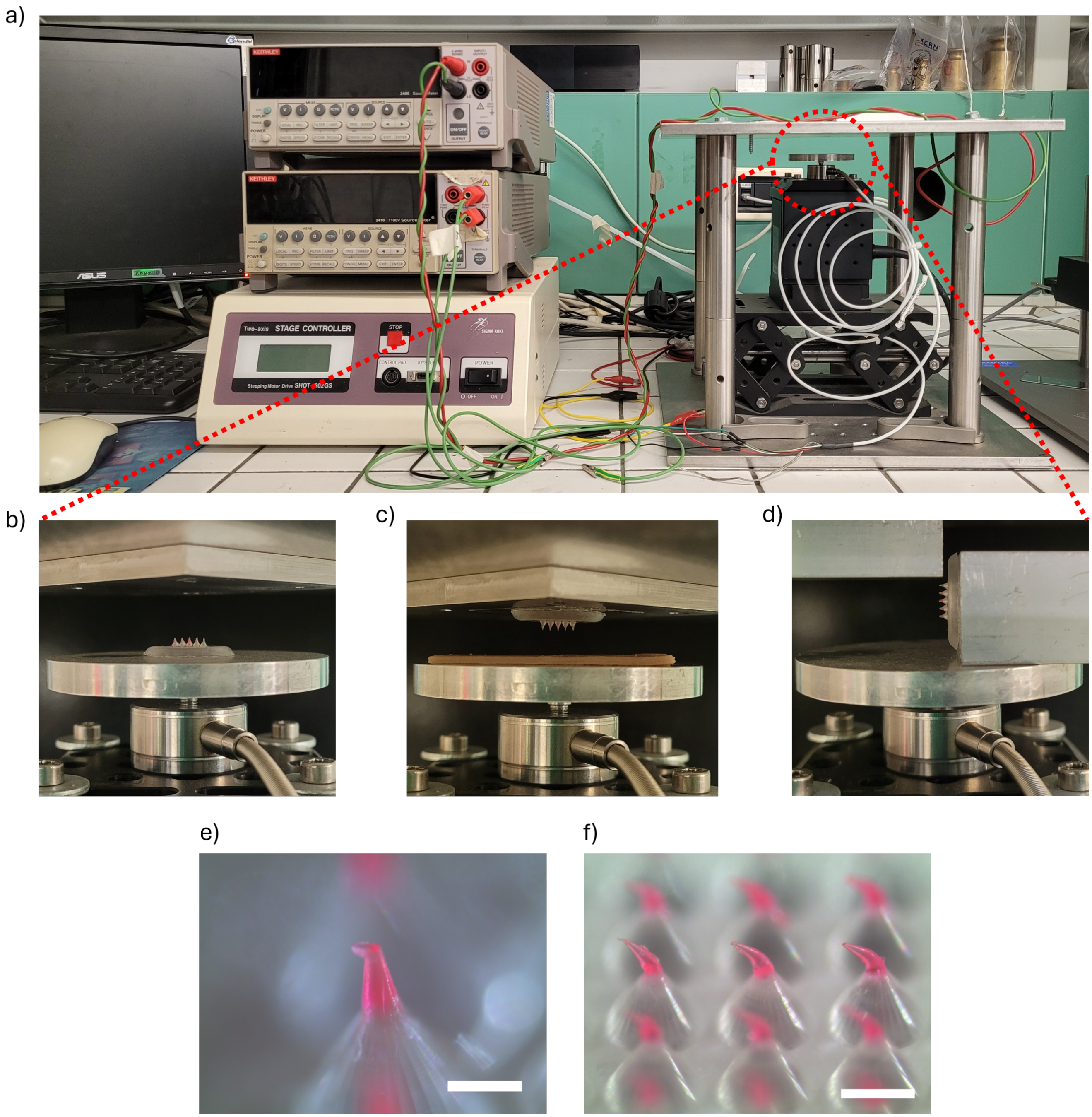

Figure S3. Mechanical setup and characterization of the QR code microneedle patches. a) Photograph of the custom LabVIEW-controlled mechanical testing setup, consisting of a 20 N load cell, a source meter unit (SMU), and a motorized translation stage. b-d) Photographs of QR-code microneedle patch used for mechanical testing: (b) compression force measurement, (c) penetration force testing with porcine skin, and (d) shear force testing setup. e, f) Bird's-eye images of microneedles without air bubble after mechanical testing, showing tip deformation after (e) compression force application (scale bar: 500 μm), and (f) shear force testing (scale bar 1 mm).

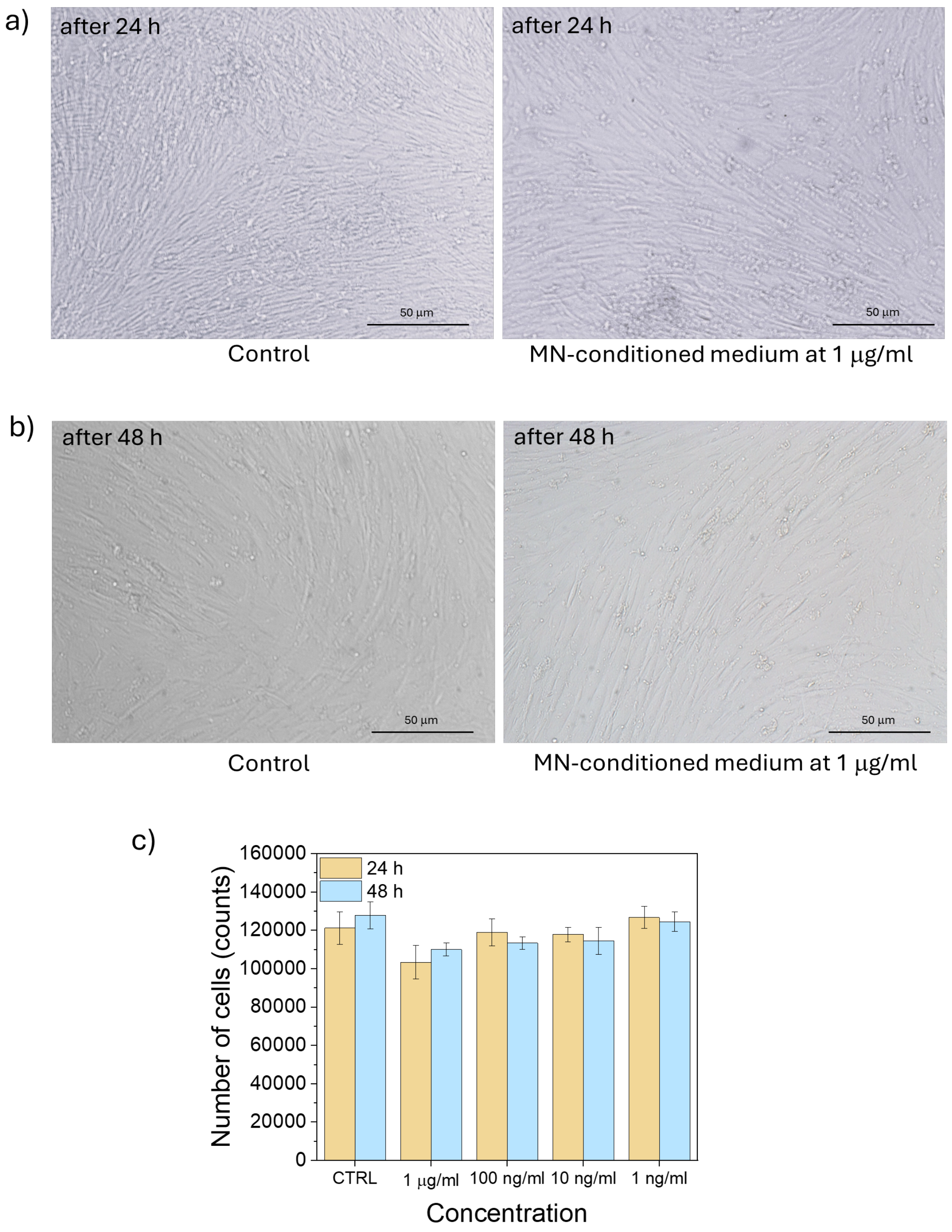

Figure S4. *In vitro* cytocompatibility assessment of microneedle extracts. Representative phase-contrast microscopy images of normal human dermal fibroblast (HDNF) cells after (a) 24 hours and (b) 48 hours exposure to microneedle (MN) extracts at the 1 µg/ml concentration. Cell morphology and confluency are comparable to control conditions, indicating no observable cytotoxic effects. Scale bars: 50 µm. c) Quantitative cell viability of HDNF cells following exposure to MN extracts at the indicated concentrations for 24 and 48 hours. Cell viability remains comparable to control levels across all conditions, indicating no significant cytotoxic effects. Data are presented as mean ± SD of three independent wells.

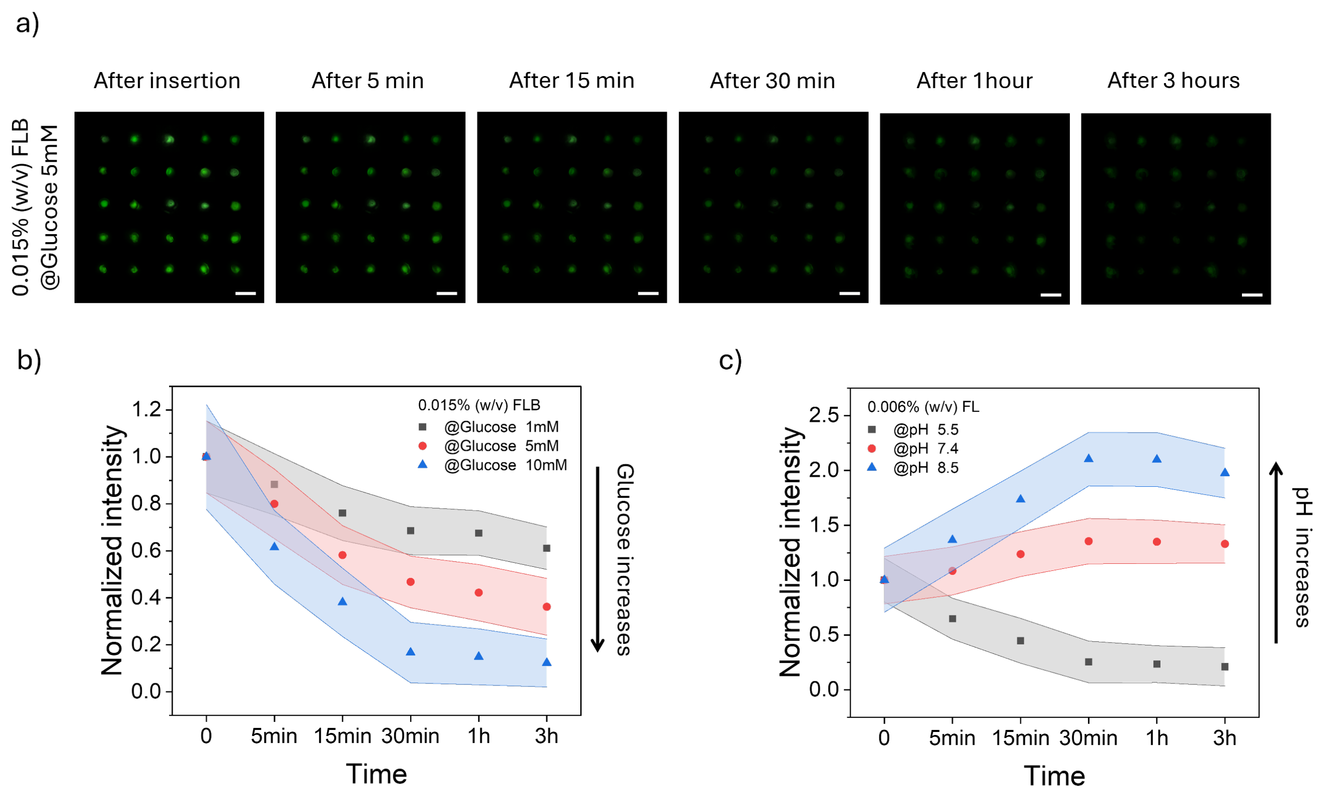

Figure S5. Time kinetics characterization of probe fluorescent response. a) Top-view fluorescence images of a microneedle patch loaded with glucose probe (0.015% (w/v) FLB) after insertion into synthetic skin preconditioned with 5 mM glucose at pH 7.4, captured at various time intervals. Scale bar 1 mm. b,c) Normalized fluorescence intensity changes over time for glucose (b) and pH (c) probes at different target concentrations, demonstrating the stabilization period required for reliable sensor readout.

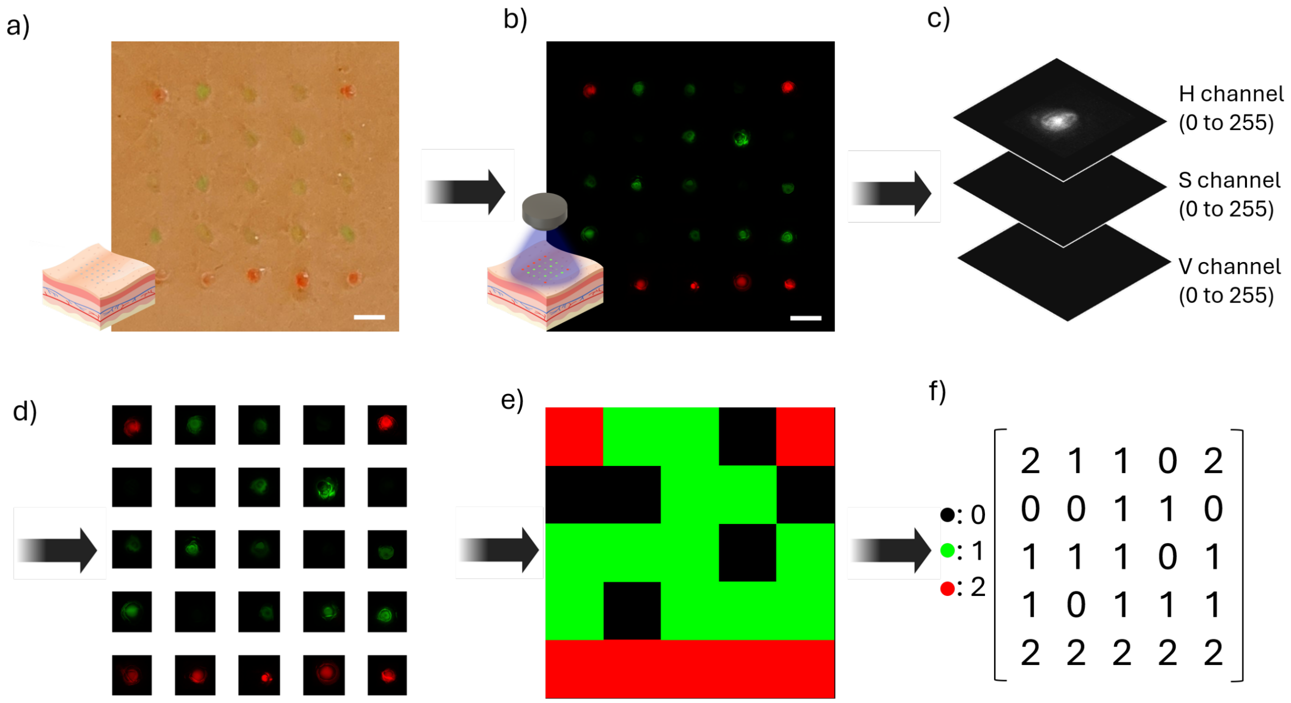

Figure S6. Decoding process for fluorescent QR code microneedle patches. (a–f) Following insertion of the microneedles into the skin (a), fluorescence images are acquired (b), each microneedle's fluorescence signal is analyzed using the HSV (Hue, Saturation, Value) color model to isolate the contribution of each fluorophore (c), then the image is segmented to isolate individual microneedle regions for conversion to a final QR-code (d). The presence or absence of a fluorescence signal is then assessed based on predefined HSV thresholds. For each color-positive microneedle above threshold, the corresponding color is assigned to generate a digital QR code image (e). Digital values were defined as follows: 0 = no signal, 1 = green fluorescence (FL or FLB), and 2 = red fluorescence (Nile Red). (f) Final binarized QR code confirming accurate encoding and signal retrieval. Scale bars are 1 mm.

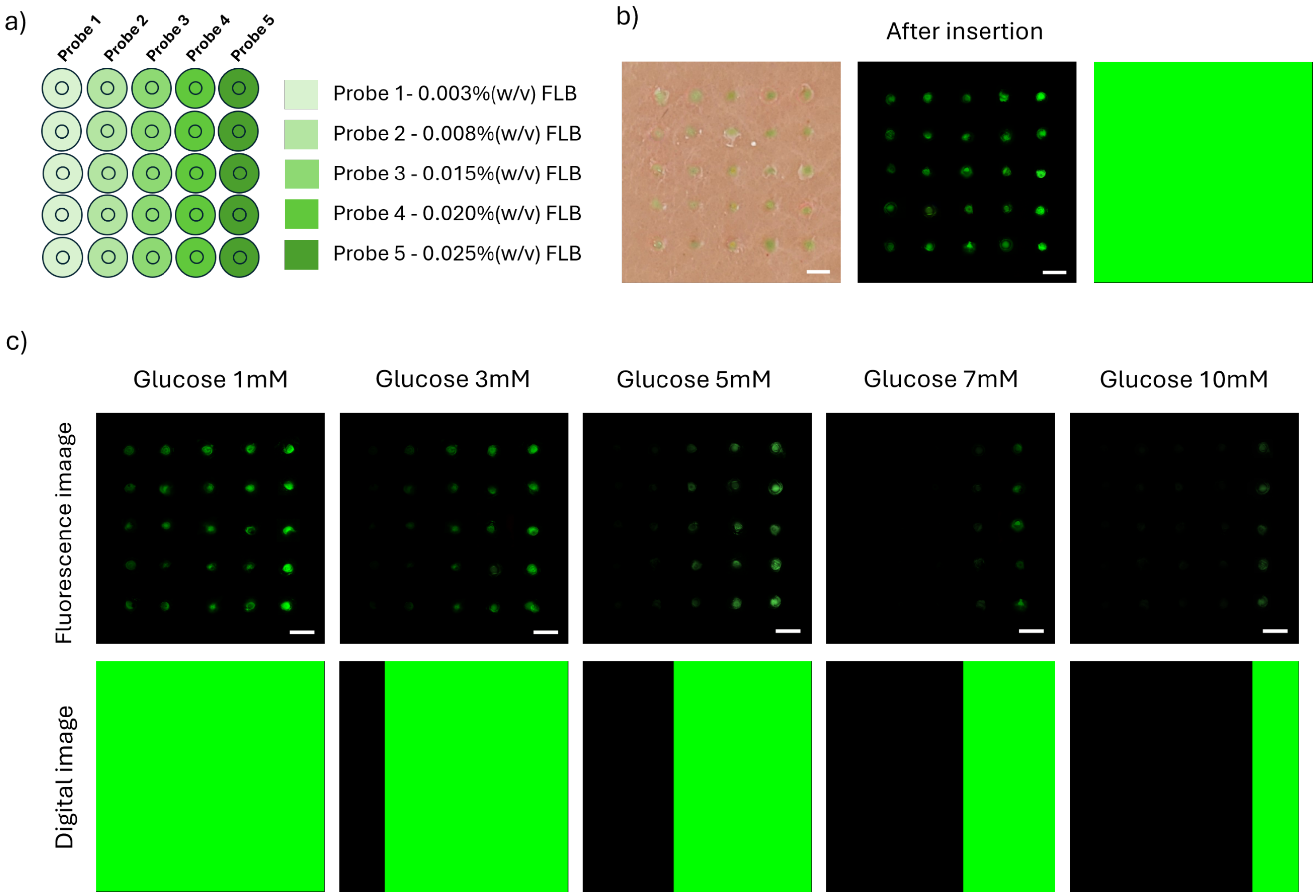

Figure S7. Optimization of FLB probe concentrations for glucose-sensitive threshold activation. a) Schematic top view of a 5×5 microneedle array containing fluorescein boronic acid (FLB) probes at different concentrations, each tuned to deactivate above specific glucose thresholds ranging from 2 to 8 mM, in 2 mM increments. b) Bright-field, fluorescence, and digitally reconstructed QR code images of the patch following insertion into synthetic skin. c) Fluorescence images of microneedle patches inserted into synthetic skin equilibrated at different glucose concentrations (1-10 mM), alongside corresponding binarized QR code outputs generated via HSV-based image processing. Scalebars are 1 mm.

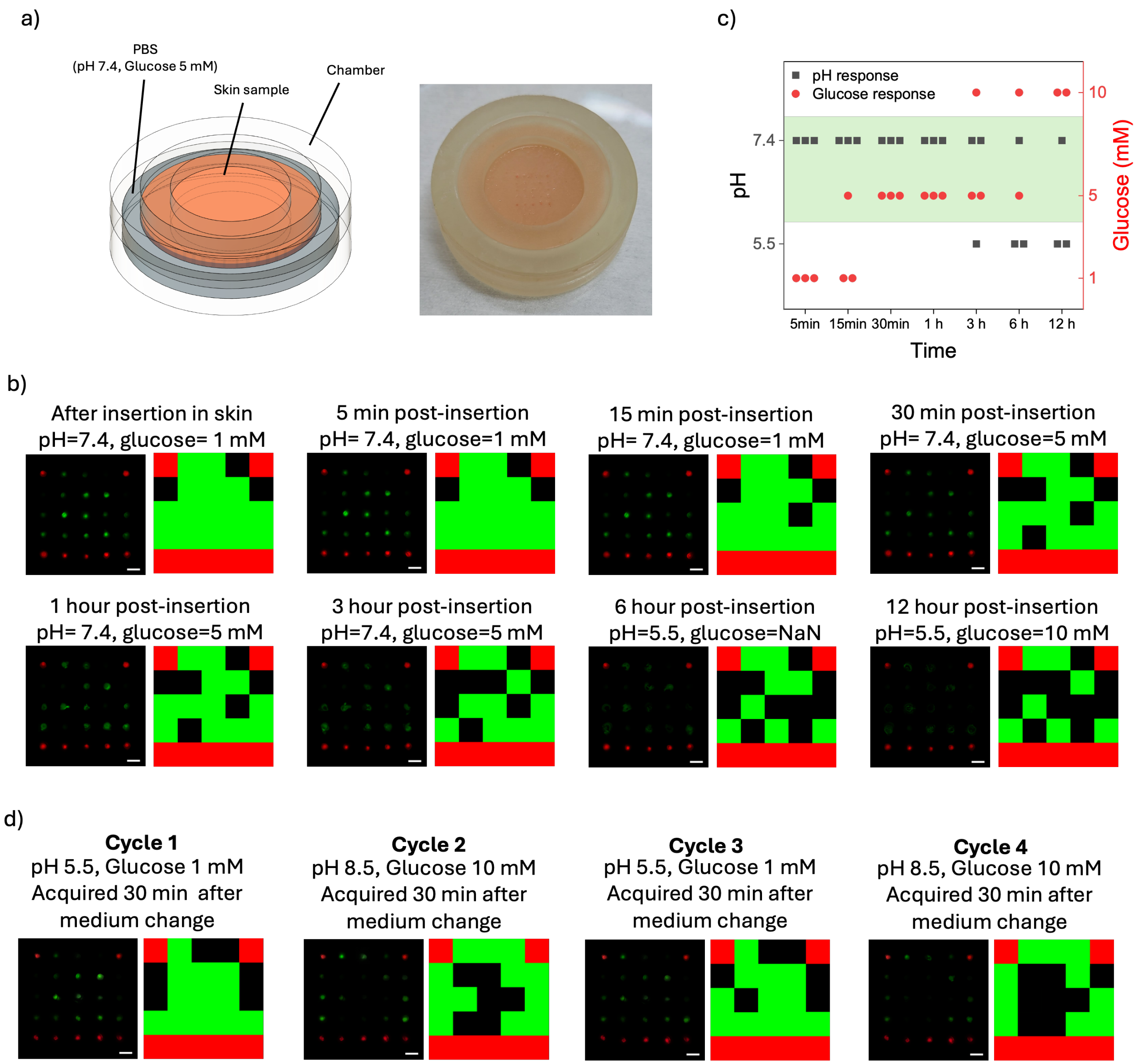

Figure S8. Reliability assessment and reversible switching behavior of QR-code microneedle patch responses under physiological and step-change analyte conditions. a) Schematic (left) and photograph (right) of the incubation test chamber consisting of a buffer reservoir and synthetic skin clamped on top. b) Fluorescence images and corresponding QR code outputs from a single microneedle patch inserted into synthetic skin and incubated at 37 °C in PBS buffer (pH 7.4, glucose 5 mM) over various time intervals. c) Final decoded responses from three different patches tested under the same conditions, highlighting the optimal operational window between 30 and 60 minutes ($n = 3$). The green-shaded area indicates time points where the QR code output correctly reflects the expected pH and glucose values. Scale bars 1 mm. d) Representative fluorescence images (left in each pair) and corresponding decoded QR-code outputs (right) from the same microneedle patch during sequential alternation between two analyte states (pH 5.5 / glucose 1 mM and pH 8.5 / glucose 10 mM) at incubated synthetic skin. The triplicate implementation of each digital "bit" provides redundancy, enabling the decoding algorithm to correctly interpret the QR-code pattern even in the presence of occasional single-

needle misclassification. Green fluorescence denotes sensing microneedles and red fluorescence denotes reference microneedles for orientation. Scale bars, 1 mm.

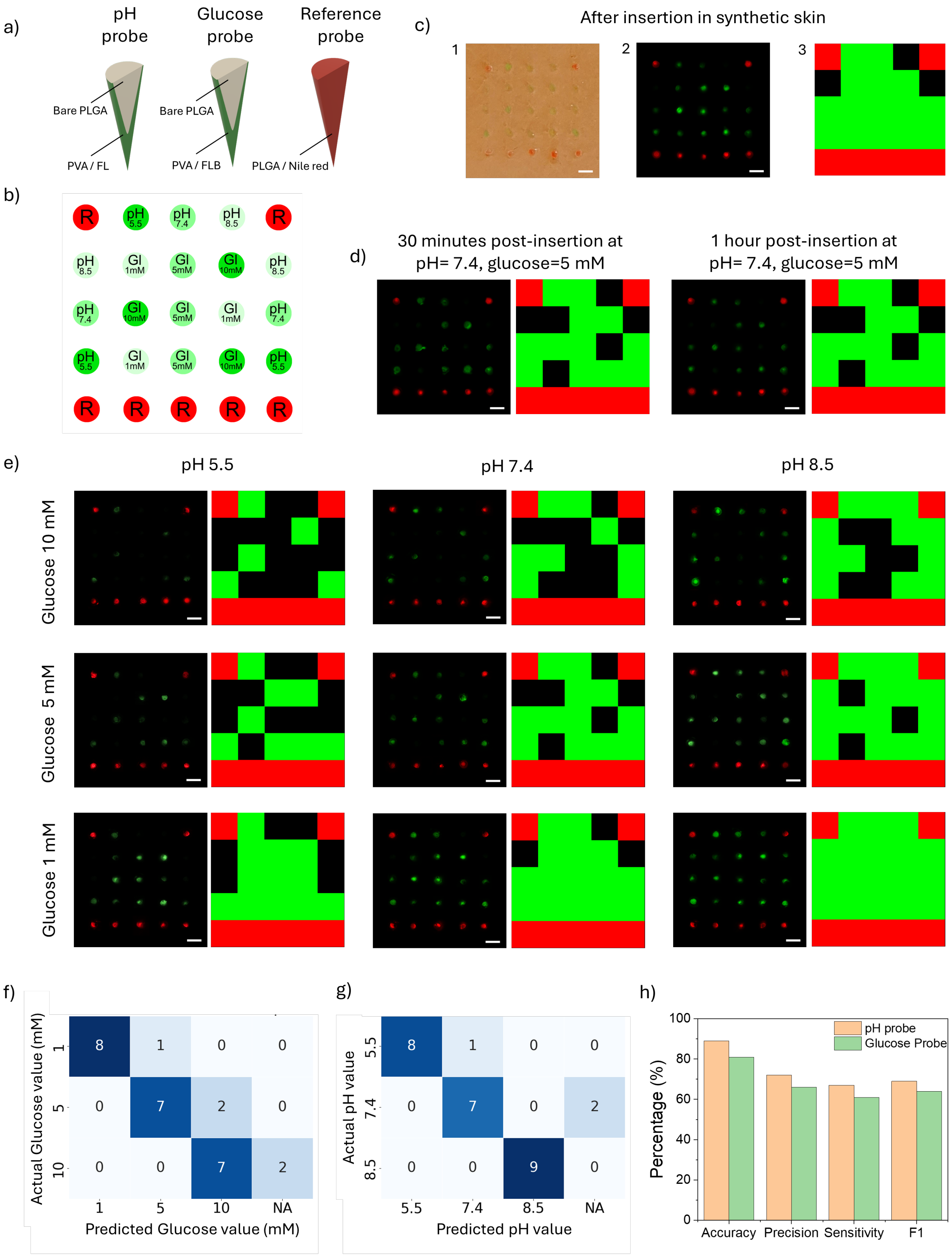
a)
pH probe
Glucose probe
Reference probe
Bare PLGA
PVA / FL
Bare PLGA
PVA / FLB
PLGA / Nile red
b)
R
pH 5.5
pH 7.4
pH 8.5
R
pH 8.5
Gl 1mM
Gl 5mM
Gl 10mM
pH 8.5
pH 7.4
Gl 10mM
Gl 5mM
Gl 1mM
pH 7.4
pH 5.5
Gl 1mM
Gl 5mM
Gl 10mM
pH 5.5
R
R
R
R
R
c)
After insertion in synthetic skin
1
2
3
d)
30 minutes post-insertion at pH= 7.4, glucose=5 mM
1 hour post-insertion at pH= 7.4, glucose=5 mM
e)
pH 5.5
pH 7.4
pH 8.5
Glucose 10 mM
Glucose 5 mM
Glucose 1 mM
f)
Actual Glucose value (mM)
1
5
10
8
1
0
0
0
7
2
0
0
0
7
2
1
5
10
NA
Predicted Glucose value (mM)
g)
Actual pH value
5.5
7.4
8.5
8
1
0
0
0
7
0
2
0
0
9
0
5.5
7.4
8.5
NA
Predicted pH value
h)
Percentage (%)
100
80
60
40
20
0
pH probe
Glucose Probe
Accuracy
Precision
Sensitivity
F1

Figure S9. Characterization of dual-analyte pH/glucose QR code microneedle patches in synthetic skin. a) Schematic of the microneedle core-shell structure for pH/glucose detection and solid PLGA reference needles. b) Patch layout with different fluorophore concentrations enabling dynamic threshold switching for pH and glucose. c) Top view of a patch after insertion into synthetic skin: bright-field image (1), corresponding fluorescence (2) and converted QR code (3). d) Time-resolved QR code output under physiological conditions (pH 7.4, glucose 5 mM) showing stabilization after 30 min and accurate readout for ~1 h. e) Representative QR codes generated from patches exposed to all pH/glucose combinations (n = 27 patches). f,g) Confusion matrices for pH and glucose classification across nine patches per condition (n = 27 patches). h) Quantitative analysis confirming robust and reproducible performance, with classification accuracies of 89% for pH and 81% for glucose (n = 27 patches). Green fluorescence indicates sensing microneedles, while red fluorescence marks reference microneedles for orientation and insertion confirmation. Scale bars:1mm.

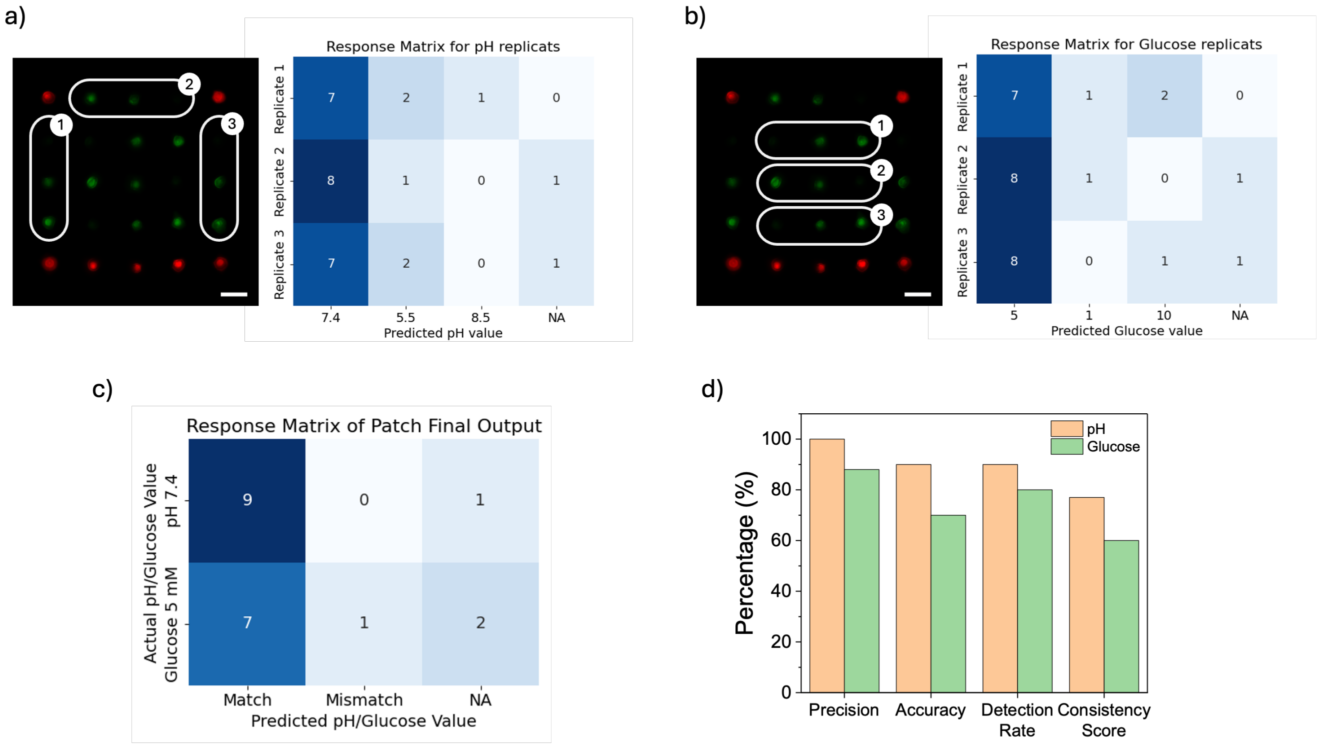

Figure S10. Reliability assessment of pH/glucose QR code microneedle patch responses under consistent pH and Glucose conditions. a) Fluorescence image showing the triplicate microneedles used for pH detection within a patch (left), and the corresponding pH replicate responses across 10 different patches under the same condition (pH 7.4, glucose 5 mM), as processed by the classification script (right).b) Fluorescence image of the triplicate microneedles used for glucose detection within a patch (left), and corresponding replicate responses across 10 different patches under the same condition, as analyzed by the script (right). c) Matrix summarizing the prediction accuracy of pH and glucose responses for 10 measurements each, under fixed conditions (pH 7.4, glucose 5 mM). d) Quantitative analysis illustrating the reliability and consistency of QR-code microneedle patch performance for both pH and glucose detection. Scale bars are 1 mm.
